# Targeting Trypanothione Synthetase and Trypanothione Reductase: Development of Common Inhibitors to Tackle Trypanosomatid Disease

**DOI:** 10.3390/ph18081182

**Published:** 2025-08-11

**Authors:** André Augusto, Inês Costa, Jaime Conceição, Maria L. S. Cristiano

**Affiliations:** 1 Faculty of Sciences and Technology, Universidade do Algarve, 8005-139 Faro, Portugal; a71282@ualg.pt (A.A.); a52917@ualg.pt (I.C.); jmconceicao@ualg.pt (J.C.); 2 Centre for Marine Sciences (CCMAR), Universidade do Algarve, 8005-139 Faro, Portugal; 3 Algarve Biomedical Centre Research Institute (ABC-Ri), Universidade do Algarve, 8005-139 Faro, Portugal; 4 Centre for Interdisciplinary Studies (CEIS20), Universidade de Coimbra, 300-548 Coimbra, Portugal

**Keywords:** neglected tropical diseases, leishmaniases, trypanosomiasis, trypanothione reductase, trypanothione synthetase, inhibitors

## Abstract

Neglected Tropical Diseases (NTDs) encompass a range of disorders, including infectious diseases caused by viruses, bacteria, parasites, fungi, and toxins, mainly affecting underprivileged individuals in developing countries. Among the NTDs, those caused by parasites belonging to the Trypanosomatidae family are particularly impacting and require attention, since the lack of financial incentives has led to constraints on the development of novel drugs to tackle them effectively. To circumvent the minor advances in drug discovery in this area, academic research emerges as a crucial player, namely through the identification and validation of new drug targets, thereby contributing to the development of more efficient, safe, and less expensive therapies against Trypanosomatidae infections. Noteworthy, this is a matter of utmost urgency since these diseases are endemic in countries with low socioeconomic standards. This review provides a comprehensive understanding of the current paradigm of NTDs caused by parasites belonging to the Trypanosomatidae family, addressing the ongoing limitations and challenges associated to the current chemotherapy solutions for these diseases and discussing the opportunities unravelled by recent research that led to the identification of new biomolecular targets that are common to Trypanosomatidae parasites. Among these, the unique properties of Trypanothione Synthetase (TryS) and Trypanothione Reductase (TryR), two key protozoan enzymes that are essential for the survival of *Trypanosoma* and *Leishmania* parasites, will be emphasised. In addition to a critical analysis of the latest advances in the discovery of novel molecules capable of inhibiting TryS and TryR, the possibility of dual targeting through a combination of TryS and TryR inhibitors will be addressed

## 1. Introduction

According to the World Health Organization (WHO), Neglected Tropical Diseases (NTDs) comprise a range of twenty diseases predominantly found in developing countries, with high incidence among marginalised populations. Globally, about 1.65 billion people are thought to contract at least one NTD, and over 35% of the worldwide NTD load is in Africa [1]. The chronic consequences from an NTD infection might include severe disability, deformity, and malnourishment. Apart from the severe impact on health, and thus on the quality of life, NTDs also contribute to economic imbalance stemming from detrimental consequences on the capacity of affected individuals to work, on children’s growth and educational attendance, and on high medication and healthcare costs [1,2].

Concerning the status of NTD pharmacotherapy and the limitations of the available treatments, current medications are often associated with toxicity and nasty side effects, require prolonged schemes of therapy, and are expensive, especially by the standards of the most affected countries. Furthermore, the development of drug resistances by NTD pathogens has led to a decrease in the efficacy of available treatments. NTD management, prevention, and surveillance are also precarious, and to worsen the situation even more, there is a notable defeasibility in the availability of certain healthcare products to people in need. Therefore, it is imperative to develop better therapeutic options to effectively decrease the spread of NTDs [2].

NTDs are endemic in countries with low socioeconomic standards, where financial constraints often compromise the access to the right treatments. This lack of profitability discourages pharmaceutical companies from investing funds in the development of new drugs [3]. Some strategies have been emerging to circumvent the lack of innovative treatments against NTDs. Collaboration between public health and governmental organisations is one of the recent tactics, and academic research plays a crucial role in this field, mostly by supporting the preclinical development of novel candidates [4].

The wide range of NTDs can be subdivided into four different subgroups: parasitic helminth NTDs; parasitic protozoan NTDs; bacterial and fungal NTDs; and viral and other kinds of NTDs [5]. This review is focused on vector-borne infections belonging to the parasitic protozoan NTD subgroup, addressing namely leishmaniases, Chagas Disease, and Human African Trypanosomiasis. These diseases are caused by the protozoans *Leishmania*, *Trypanosoma cruzi*, and *Trypanosoma brucei*, respectively, which belong to the *Trypanosomatidae* family. There are common and unique features among these parasites that are worth emphasising, namely their genome conservation and architecture, amino acid identity, and the existence of unique subcellular structures, including glycosomes and kinetoplast [6]. Noteworthy, such similarities could create opportunities for developing compounds capable of addressing a conserved biomolecular target shared by this family of parasites, thus enabling the development of a common inhibitor capable of treating infections by parasites across the *Trypanosomatidae* family. In this context, trypanothione reductase (TryR) raised attention due to its vital role in maintaining parasites’ redox homeostasis, by reducing the disulphide bridge of trypanothione disulphide (TS_2_) to reduced trypanothione T(SH)_2_, which is responsible for the elimination of reactive oxygen species (ROS) that are toxic to the parasites. Moreover, TryR is not present in the mammalian host, which, instead, possesses glutathione reductase (GR). All these features support the interest in TryR as a new drug target, and the development of suitable TryR inhibitors could efficiently and specifically hamper the redox homeostasis system among all Trypanosomatidae. Indeed, all enzymes associated to the redox machinery overarching these parasites are considered promising candidates for target-based drug design and development. In addition to TryR inhibitors, the latest advances in the development of novel inhibitors of trypanothione synthetase (TryS), an enzyme involved in the synthesis of the dithiol T(SH)_2_, will also be addressed. Additionally, the combined use of different inhibitors that act on different reaction steps of the same metabolism pathway could lead to synergetic therapeutic effects and reduce the risk of developing drug resistance.

## 2. Trypanosomatid Diseases

Leishmaniases are a group of zoonotic diseases caused by protozoa of the genus *Leishmania*, disseminated through the bites of infected female sandflies (*Phlebotomus spp.*) [7]. While several animals can be exposed to this parasite, humans and dogs are the most affected ones. Leishmaniases broadly encompass three major syndromes, known as cutaneous leishmaniasis (CL), mucocutaneous leishmaniasis (MCL), and visceral leishmaniasis (VL) [8]. Among all forms, VL is the most dangerous one, being potentially fatal in the absence of appropriate treatments [9]. In general, most infections remain asymptomatic (around 65%) [10]. However, when the host becomes symptomatic, leishmaniasis can be widely heterogeneous in terms of severity, ranging from self-resolving skin ulcers (CL), to life-threatening illness (VL) [11]. VL is characterised by a number of symptoms, such as fever, weight loss, splenomegaly, variable hepatomegaly, pancytopenia, elevated liver enzymes, and blood abnormalities [12,13]. Regarding MCL, even though it is depicted as less hazardous, it is still considered the most disfigurative leishmaniasis syndrome, due the destructive mucosal lesions that it causes. Moreover, MLC may arise from inadequately treated CL infection [14]. Leishmaniases remain a significant public health problem across eighty eight endemic countries (mainly in South and Central America, Africa, Asia, and Southern Europe) [15]. Recent outbreaks in other geographical regions (e.g., northern latitudes and higher altitudes in Italy, the Pyrenees, and Germany) have also occurred and are linked to multiple factors, such as the dispersion of *Phlebotomus* sandflies due to climate change and the scattering of infected dogs through adoption services [16]. Chagas Disease, also known as American Trypanosomiasis, is an infectious disease provoked by the protozoan *T. cruzi* that affects various mammalian species (e.g., humans, horses, pigs, cats, and dogs) across America. From the estimated 6–7 million cases of Chagas Disease globally, around six million are found in this region [17]. *T. cruzi* transmission mainly occurs through contact of humans with faeces or urine of infected triatomine bugs (also known as kissing bugs), but it can also spread via blood transfusion, through organ transplant, and congenitally [18]. Indeed, it is anticipated that 8600 babies will contract the infection during gestation [19]. Clinical manifestations range from mild to severe symptoms, including digestive issues like dysphagia, oesophageal reflux, and regurgitation, among other oesophageal motility disturbances, which may vary between mild achalasia to megaoesophagus [20]. The increase in travelling and migration between endemic and non-endemic areas has created some epidemic focus points in Europe and the United States, raising concerns about the disease’s control in the future [21]. Other factors that have also influenced the epidemiologic status of Chagas Disease are the international interchange of live animals and climate change [19].

Human African Trypanosomiasis, also denominated as Sleeping Sickness, is a parasitic infectious disease caused by the protozoans *T. brucei gambiense* and *T. brucei rhodesiense*. Due to strong efforts focused on controlling this disease, the incidence of Human African Trypanosomiasis has drastically decreased, with only 837 reported cases in 2022 [22]. Nevertheless, it remains a serious health problem due to its near-100% mortality rate in untreated or inadequately treated cases [23]. It is solely transmitted through the bite of infected bloodsucking flies of the genus *Glossina*, also designated tsetse flies, endemically found in sub-Saharan Africa [24]. The rapid spread rate of Sleeping Sickness has led to the emergence of confirmed reported cases of tourists infected with *T. brucei rhodesiense* while visiting areas in eastern and southern Africa, while some cases of *T. brucei gambiense* have been detected in non-endemic countries, mainly among western and central African migrants [25]. Sleeping Sickness cluster of symptoms in the early stage of the disease may include fever, joint pain, and swollen lymph nodes. The progression to a late stage is accompanied by injuries in the central nervous system (CNS), triggering the onset of sleep disturbances (the reason why it is called the Sleeping Sickness), neurological and psychiatric disorders, and coma [26]. Eventually, in the worst case, this infection can culminate in death if left untreated [27].

## 3. The Current Treatments for Infections Caused by Trypanosomatidae Parasites

**Figure 1:**
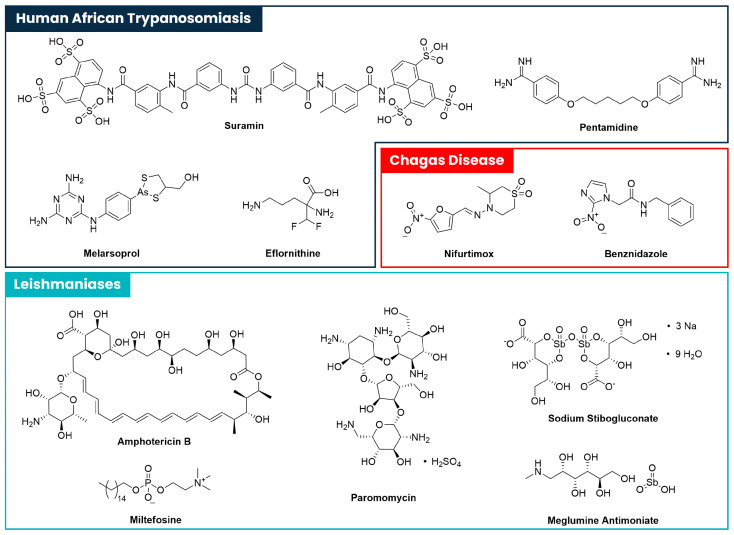
Representation of the chemical structures of the available drugs used in the treatment of Human African Trypanosomiasis (highlighted in dark blue), Chagas Disease (highlighted in red), and leishmaniases (highlighted in light blue). Molecular structures are of public domain and were drawn using the available software Chemdraw Ultra 12.0.

Even though leishmaniases, Chagas Disease and Human African Trypanosomiasis are serious health conditions that can be fatal if left untreated, the available therapeutic arsenal to tackle these parasitic infections presents several liabilities and is sometimes ineffective. The chemical structures of the drugs currently in use for the treatment of those diseases are represented in Figure 1, and their pharmacological and pharmacotherapeutic properties are discussed briefly below.

Suramin (Figure 1) is a phenylurea derivative comprising carboxamide and naphthalenesulfonic acid moieties. This drug is used to treat various health conditions, due to its broad pharmacological activity. Although its mechanism of action remains unknown, the molecule is believed to interact with several parasitic enzymes involved in energy metabolism [28]. Suramin is used in the treatment of acute Human African Trypanosomiasis. Since it cannot cross the blood–brain barrier, it is only suitable for use during the first stage of infection, when the disease has not yet progressed to the CNS, requiring early diagnosis, which is often compromised by the low availability of healthcare services in endemic regions. In addition, the standard treatment regimen of Suramin is complex and needs to be followed by qualified healthcare professionals, since Suramin is associated with high risk of toxicity. Possible undesirable effects include nephrotoxicity, hypersensitivity reactions, dermatitis, anaemia, peripheral neuropathy, and bone marrow toxicity [29,30].

Pentamidine (Figure 1) is a diether derived from pentane-1,5-diol, comprising terminal 4-amidinophenyl groups. It is used to treat early-stage infections caused by *T. brucei gambiense*. Similar to Suramin, its effectiveness relies on early diagnosis. Studies on its mechanism of action revealed that it critically interferes with the synthesis of deoxyribonucleic acid (DNA) through the formation of cross-links in adenine-thymine-rich regions. Pentamidine has also shown to interfere with ribonucleic acid (RNA), phospholipids, proteins, and mitochondrial type II topoisomerases [31]. The treatment regimen is also quite complex, requiring the administration of 7–10 intramuscular injections or intravenous infusions of 2 h [32]. Therefore, patients need to be constantly followed by healthcare professionals for assistance in the administration, and also to control any kind of adverse reactions that may arise from the use of Pentamidine [30]. Some of the most frequent adverse reactions include hypoglycaemia, hypotension, nausea, and vomiting. Nephrotoxicity, acute pancreatitis, allergic reactions, and cardiac arrhythmias may also occur in rare cases [33]. It is important to emphasise that this drug can be used as an alternative treatment for *Leishmania* spp. infections [34].

Melarsoprol (Figure 1) is a trivalent organic arsenical compound used for the treatment of Human African Trypanosomiasis [35] in the second stage of infection, when the CNS is already affected [36]. The treatment involves a complex 10-day regimen, with daily dosages of 2.2 mg/kg through intravenous injections [35,37]. Melarsoprol is a highly toxic substance, causing a severe encephalopathic syndrome in 1.5% to 28% of treatments [38]. In addition to its high toxicity, complex treatment regimen, and route of administration, Melarsoprol is also highly susceptible to drug resistance. As such, it should only be handled by qualified healthcare professionals.

Eflornithine (Figure 1) is an ornithine amino acid derivative comprising a difluoromethyl group at position 2, used as a first-line drug against second stage Human African Trypanosomiasis [39]. Eflornithine is known to irreversibly inhibit ornithine decarboxylase, an enzyme that converts ornithine into putrescine, which is then converted into spermidine upon reaction with decarboxylated S-adenosylmethionine (SAM), a metabolic step that is catalysed by spermidine synthase [40,41]. Spermidine is an important precursor of trypanothione. Eflornithine is an expensive drug and is administered in a complex and intensive treatment regimen [39]. Although it is better tolerated than Melarsoprol, its most common adverse reactions may include temporary hearing loss, otitis media, pyrexia, pneumonia, and diarrhoea [42]. All aspects considered, it becomes clear that the main challenges with this drug are its long and complex treatment regimen and high cost.

Eflornithine can also be combined with Nifurtimox, mentioned below, to increase efficacy against second stage Human African Trypanosomiasis [43], reducing the number of administrations and the treatment cost, when compared to Eflornithine in monotherapy [30]. Even though simpler, the therapeutic regimen still needs to be followed by healthcare professionals due to the need of Eflornithine long intravenous infusions [39].

Nifurtimox (Figure 1) is a nitrofuran derivative used to treat Chagas Disease [44]. Its therapeutic regimen for individuals weighting 40 kg or more is made through the oral route, for a total of sixty days [18]. Nifurtimox is believed to induce oxidative stress inside the parasites through the production of ROS [45,46]. The most observed adverse reactions include weight loss, irritability, sleepiness, and other nervous system manifestations. Depression, peripheral neuropathy, psychiatric symptoms, rash, pruritus, and drug-associated hepatitis may also occur [47]. Although Nifurtimox is a reasonably effective drug against Chagas Disease, the problems associated with its tolerability and patient compliance should be taken into account [30].

Benznidazole (Figure 1) is a nitroimidazole derivative bearing a benzyl amide moiety, used as alternative treatment of infections caused by *T. cruzi* [48]. Although its mechanism of action is not yet fully understood, it is believed to be a prodrug bioactivated by some type I trypanosomal nitroreductases, a group of enzymes only expressed in protozoan parasites. Through a stepwise mechanism, benznidazole is reduced into the corresponding hydroxylamine, which is then converted into a dialdehyde glyoxal by several nonenzymatic transformations. This highly reactive metabolite binds to proteins, lipids, DNA/RNA, and small molecules, blocking the synthesis of new DNA strands and inhibiting the *T. cruzi* antioxidant system, rendering the parasite highly susceptible to oxidative damage [49]. Benznidazole is administrated orally during thirty to eighty days of treatment [50]. Although it is quite effective against Chagas Disease, its toxicity compromises patients’ compliance to the treatment regimen [30]. Common undesirable effects include dermatological and digestive problems and neuropathy, and, though more rarely, myelotoxicity, hepatotoxicity, arthralgia, and increased creatinine levels may also be observed [51].

Amphotericin B (Figure 1) is an antifungal drug that can be used in the treatment of VL [52]. It works by binding to ergosterol, an essential steroid abundant in fungal and some parasitic cell membranes, consequently creating transmembrane pores that disrupt ionic balance and kill the organism [53]. Amphotericin B is an expensive drug, is administered intravenously, and its dosage must be adjusted to each patient, given its low therapeutic index and high toxicity, mainly manifested in the form of nephrotoxicity [54].

Miltefosine (Figure 1) belongs to a class of organic molecules known as phosphocholines and is used in the treatment of infections caused by *Leishmania* parasites [55]. Its mode of action is not yet fully understood, although several authors reported that Miltefosine interferes with the lipid-dependent signalling pathways [56], inhibits cytochrome C oxidase [57], induces apoptosis-like cell death [58], and can also disrupt the Ca^2+^ homeostasis inside the parasitic cell, leading to apoptosis or necrosis of trypanosomatids [59,60,61]. Unlike the previous drugs, the oral treatment regimen with Miltefosine is quite simple and practical for patients suffering from this disease. However, patient compliance should be monitored [62]. Additionally, Miltefosine is highly teratogenic, which limits its use by pregnant women infected with Leishmaniasis [30].

The pentavalent antimonials sodium stibogluconate and meglumine antimoniate (Figure 1) are antimony complexes frequently used in the treatment of leishmaniases [30]. Both molecules show similar efficacy and toxicological properties [63]. Their mechanism of action remains elusive, although two main models were considered: the prodrug model proposes that pentavalent antimonials are reduced within the organism into more toxic and active trivalent antimonial compounds, inhibiting the reduction of TS_2_ and the expression of essential genes needed for the parasite survival [64]; the other model assumes that pentavalent antimonials have intrinsic anti-leishmanial activity. Namely, they were ascribed to inhibit the type I DNA topoisomerases of the parasite [65,66], to be directly involved in adenosine diphosphate (ADP) and guanosine diphosphate (GDP) reduction [67], and to obstruct major energy-driven metabolic pathways, as fatty acid-oxidation, glycolysis, and ADP phosphorylation, inducing oxidative stress [68]. The treatment regimen involves a daily dose of 20 mg/kg/day, for 20 to 30 days, through intramuscular or intravenous administration [30,69]. The parenteral route of administration, the increasing development of resistances, and the high toxicological profile represent the main limitations associated to the use of pentavalent antimonials [30]. Some of the most common undesirable effects include musculoskeletal pain, headache, nausea, and asthenia. Rarer undesirable effects are cardiotoxicity, hepatotoxicity, nephrotoxicity, and pancreatitis [70].

Paromomycin (Figure 1) is an aminoglycoside often used in the treatment of VL. Its mechanism of action against *Leishmania* is not fully understood. Several authors proposed that this drug inhibits the translocation and recycling of ribosome subunits and that it also inhibits a parasite’s protein synthesis through modifications in the mitochondrial membranes [71,72,73,74,75,76,77,78,79]. As for most of the other drugs mentioned previously, a major weakness of Paromomycin is its complex treatment regimen, which involves a daily intramuscular dosage of this drug for twenty-one days. Paromomycin can cause severe side effects, including ototoxicity and nephrotoxicity, which is why its usage has to be followed by qualified healthcare professionals [71].

The main aspects of each drug used to treat NTDs caused by *Trypanosomatidae* parasites are summarized in Table 1.

**Table 1:** Main features of the therapeutic arsenal available for the treatment of NTDs caused by Trypanosomatidae parasites.

Drug	Mechanism of Action	Pharmaceutical Dosage Form(s)	Route(s) of Administration	Main Therapeutic Indication(s)	Pathogen	Undesirable Effects	Contraindications/ Precautions	Limitations	References
Suramin	Thought to involve the inhibition of several cellular enzymes (e.g., glycerol-3-phosphate oxidase and dehydrogenase)	Injectable solution	Intravenous bolus	Human African Trypanosomiasis (1st stage of infection)	*T. brucei rhodesiense*	Nephrotoxicity, hypersensitivity reactions, dermatitis, anaemia, peripheral neuropathy, and bone marrow toxicity	Avoid use in renal or hepatic impairment	Complex treatment regimen and high toxicity	[80,81,82]
Pentamidine	Inhibits DNA synthesis through formation of cross-links between two adenines. Interferes with RNA, phospholipids, and proteins essential to the parasite’s survival, including type II topoisomerase	Powder for injectable solution	Intravenous infusion and intramuscular injection	Human African Trypanosomiasis (1st stage of infection)	*T. brucei gambiense*	Hypoglycaemia, hypotension, abscess in the site of injection, nausea, vomiting, nephrotoxicity, acute pancreatitis, hyperglycaemia, hypoglycaemia, hypocalcaemia, allergic reactions, and cardiac arrhythmias	Concomitant use with nephrotoxic drugs or with other drugs that prolong QT interval, and during pregnancy and breastfeeding	Complex treatment regimen, and high toxicity	[83,84,85]
Melarsoprol	It is proposed that, upon bioactivation into melarsen oxide, it can either inhibit pyruvate kinase or form a stable adduct with trypanothione that inhibits TryR	Injectable solution	Intravenous bolus	Human African Trypanosomiasis (2nd stage of infection)	*T. brucei rhodesiense*	Reactive encephalitis, agranulocytosis, peripheral neuropathy, cardiac arrhythmias, and hypertension	Individuals with glucose-6-phosphate dehydrogenase deficiency	Complex treatment regimen, high toxicity, and susceptibility to drug resistance	[86,87,88]
Eflornithine	Irreversible inhibitor of ornithine decarboxylase	Injectable solution and tablets	Intravenous infusion and oral administration	Human African Trypanosomiasis (2nd stage of infection)	*T. brucei gambiense*	Temporary hearing loss, otitis media, pyrexia, pneumonia, diarrhoea, seizures, hepatotoxicity, and mild reversible bone marrow toxicity	Monitor potential foetal harm, monitor hearing before and during treatment, perform blood counts to avoid potential myelosuppression, and perform liver function tests due to the risk of hepatotoxicity	Complex treatment regimen and it is very costly	[89,90,91]
Nifurtimox	Involves the production of nitro-anion radicals, which, in the presence of oxygen, prevent the parasite from detoxifying ROS	Tablets	Oral administration	Chagas Disease	*T. cruzi*	Anorexia, weight loss, irritability, sleepiness, depression, peripheral neuropathy, psychiatric symptoms, other nervous system manifestations, rash, pruritus, and drug-associated hepatitis	Alcohol consumption during treatment, presence of neurological disorders, presence of hepatic and renal failure conditions, and pregnancy	Low absorption rate when taken without food and high toxicity	[92,93,94]
Benznidazole	Prodrug. Upon activation inhibits the synthesis of DNA and disrupts the parasite’s antioxidant system	Tablets	Oral administration	Chagas Disease	*T. cruzi*	Rash, pruritus, epigastralgia, nausea, vomiting, somnolence, headache, paraesthesia, neutropenia, and hypertransaminasemia	Patients who have taken disulfiram within the last 2 weeks, patients with Cockayne syndrome, alcoholic beverage consumption during and for at least 3 days after therapy, advanced cardiac progression, hepatic or renal complications, and pregnancy	High toxicity	[95,96,97]
Amphotericin B	Binds to ergosterol, causing depolarisation and increased membrane permeability, leakage of cell contents, and cell death	Powder to be dispersed for infusion	Intravenous perfusion	Visceral Leishmaniasis	*Leishmania* spp.	Nausea, vomiting, rigors, fever, hypertension or hypotension, hypoxia, and nephrotoxicity	Concurrent use with other nephrotoxic medications may enhance potential for drug-induced renal toxicity (intensive monitoring of renal function is recommended in patients requiring any combination of nephrotoxic medications)	Complex treatment regimen, high toxicity, low therapeutic index, and it is very costly	[98,99,100]
Miltefosine	Causes impairment of phospholipids biosynthesis, interferes with lipid-dependent signalling pathways, inhibits cytochrome C oxidase, induces apoptosis-like cell death, and disrupts the Ca^2+^ homeostasis inside the parasitic cell	Capsules	Oral administration	Visceral and Cutaneous Leishmaniasis	*Leishmania* spp.	Nausea, vomiting, loss of appetite, diarrhoea, increase serum creatinine, and elevated alanine aminotransferase and aspartate aminotransferase levels	Pregnancy and Sjögren-Larsson syndrome	Low absorption rate when taken without food, low efficacy, and teratogenic	[101,102,103]
Pentavalent antimonials	Inhibit the reduction of trypanothione, causing oxidative stress. May obstruct major energy-driven metabolic pathways, inducing oxidative stress, causing DNA fragmentation, and inducing apoptotic cell death through inhibition of type I DNA topoisomerase	Injectable solution	Intravenous and intramuscular injection	All forms of leishmaniases	*Leishmania* spp.	Anorexia, nausea, vomiting, abdominal pain, metallic taste in mouth, arthralgias, myalgias, acute pancreatitis, hepatitis, arrhythmias, and QT-interval prolongation	Significant renal impairment, heart failure, hepatic failure, and patients older than 55 years (sodium stibogluconate)	Complex treatment regimen, susceptibility to drug resistance, and high toxicity	[104,105,106]
Paromomycin	Inhibits protein synthesis, disrupts mitochondrial membranes and binds to the negatively charged leishmanial glycocalyx, primary lipophosphoglycan, interacting with the phosphate (–PO_4_^2−^) moiety and acidic sugars (e.g., glucuronic acid) via carboxyl (–COO^−^) groups.	Injectable solution	Intramuscular injection	Visceral Leishmaniasis	*Leishmania* spp.	Gastrointestinal hypermotility, nausea, diarrhoea, abdominal cramps, headache, vertigo, vomiting, abdominal pain, skin rash, and nephrotoxicity	Patients with intestinal obstruction	Complex treatment regimen	[75,107,108]

## 4. Trypanothione Synthetase vs. Trypanothione Reductase

Although *Leishmania* and *Trypanosoma* parasites belong to different genera, their phylum, class, and even family are the same (Euglenozoa, Kinetoplastida, and Trypanosomatidae, respectively) [109,110]. Therefore, these parasites share many common features such as gene conservation, genome architecture, protein amino acid identity, and the presence of subcellular structures (e.g., glycosomes and the kinetoplastid). Such similar taxonomical characteristics created numerous opportunities to design and develop drugs that can be more than species-specific [6]. Moreover, many of their metabolic pathways and cellular functions diverge considerably from those of other eukaryote organisms, raising the chances to develop new drugs with lower rates of adverse reactions to their users.

One of the common mechanisms is associated to the host immune response to infections caused by these parasites. To circumvent the parasite inoculation, the host’s immune cells, such as macrophages and neutrophils, release reactive oxygen species (ROS) and reactive nitrogen species (RNS) that are toxic to the parasites. Moreover, temperature boost, pH, and nutritional alterations occurring during the parasite shift from the vector to the mammalian host and inside the host also contribute to the augmentation of ROS levels. Once within the host, some cells produce oxidative micro-areas that impair the antioxidant competence of trypanosomatids. Overall, to survive in such harsh conditions, trypanosomatids must adjust their metabolism and redox homeostasis in function of the host’s over-nutritive conditions. Without a proper anti-oxidative machinery, the cell damage created by ROS will lead to the parasite’s death, pointing out the relevance of that system in the parasites’ ability to survive inside mammals [111,112].

**Figure 2:**
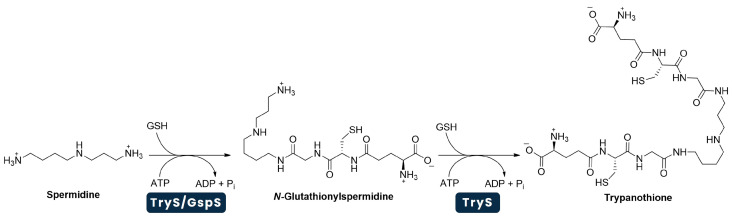
Biosynthesis of trypanothione [T(SH)_2_], occurring in two consecutive steps. In the first, driven by glutathionyl-spermidine synthetase (GspS) or TryS, the glycine carboxylate group of glutathione (GSH) is covalently bound to one of the terminal amino groups of spermidine; the second can only be catalysed by TryS, in which the glycine carboxylate group of another glutathione is covalently linked to the free terminal amino group of N-glutathionylspermidine. The whole process consumes two molecules of adenosine triphosphate (ATP) to produce one molecule of T(SH)_2_. Abbreviations: ADP, Adenosine Diphosphate; P_i_, Inorganic Phosphate. Adapted from [112,113].

**Figure 3:**
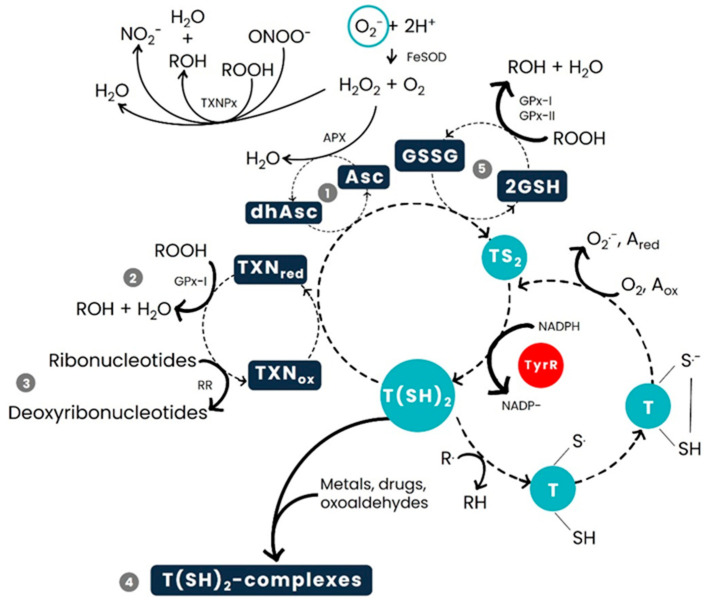
General scheme depicting trypanothione-dependent redox reactions. The process of T(SH_)2_ reduction is depicted in light blue: Whenever a trypanothione molecule complexes with an oxidative substance (e.g., metals, drugs, and oxoaldehydes), there is formation of a disulphide bridge between the two sulphurs present in each glutathione molecules (cysteine residues), with formation of a trypanothione disulphide (TS_2_). It is possible to recover the initial reduced form of trypanothione [T(SH)_2_] by breaking the disulphide bond, a reaction catalysed by TryR (red). Thus, T(SH)_2_ assumes five functions, depicted in dark blue: (1) depletion of hydrogen peroxide (H_2_O_2_) via ascorbate-dependent detoxification; (2) neutralisation of hydroperoxides (ROOH) through the complex tryparedoxin/tryparedoxin peroxidase I (TXN/TXNPx); (3) reduction of ribonucleotides into deoxyribonucleotides as essential precursor subunits of DNA; (4) elimination of metals and drugs by complexing with them and forming thiol-conjugates that are then exported/sequestered; and, finally, (5) direct reduction of disulphides into their respective thiols by the dithiol. The solid arrows represent all processes involving H_2_O_2_, the bold solid arrows indicate all initial/final substrates, and the dashed arrows illustrate all oxidation/reduction reactions. Abbreviations: A, one-electron oxidant; APX, Ascorbate-dependent Peroxidase; Asc, Ascorbate; dhAsc, Dehydroascorbate; FeSOD, Iron-Superoxide Dismutase; GPx-I, GPx-II, Glutathione Peroxidase-like tryparedoxin peroxidases I and II; GSSG, Glutathione Disulphide; GSH, Glutathione; RR, Ribonucleotide Reductase; TXNPx, Tryparedoxin Peroxidases. Adapted from [115].

The trypanothione’s metabolic pathway can be divided into two different groups of reactions, the first group leading to the formation of trypanothione (Figure 2), while all the other reactions are related to the complex redox cycle of this same molecule (Figure 3). In all these reactions, the parasites need to utilise a number of enzymes that are unique to these microorganisms, which may serve as new potential targets in the development of new drugs capable of treating the parasitic diseases they cause [112].

The reduced form of trypanothione, T(SH)_2_, assumes five major functions (Figure 3): (I) depletion of hydrogen peroxide (H_2_O_2_) via ascorbate-dependent detoxification; (II) neutralisation of hydroperoxides (ROOH) through the complex tryparedoxin/tryparedoxin peroxidase I (TXN/TXNPx); (III) reduction of ribonucleotides into deoxyribonucleotides as essential precursor subunits of DNA; (IV) elimination of metals and drugs by complexing with them and forming thiol-conjugates that are then exported/sequestered; and, finally, (V) direct reduction of disulphides into their respective thiols by the dithiol [114]. Specifically, T(SH)_2_ restores the levels of GSH by reducing glutathione disulphide (GSSG), the result of GSH’ detoxifying hydroperoxide derivatives by direct scavenging or as a substrate for glutathione peroxidase. T(SH)_2_ plays a crucial role in all the above referred processes by specifically recycling tryparedoxin (TXN) to its reduced form, dehydroascorbate (dhAsc) to ascorbate (Asc), and GSSG to glutathione (GSH). The engagement of T(SH)_2_ in all mentioned redox routes leads to its conversion into oxidized trypanothione TS_2_ that is subsequently reconverted into T(SH)_2_ by the NADPH-dependent flavoenzyme TryR (Figure 3, light blue and red) [114].

Along the trypanosomatid redox pathway, parasites need to employ several enzymes that are specific to these microorganisms, making them new potential therapeutic targets for developing new anti-parasitic drugs [112]. Among all enzymes we highlight TryS and TryR, since they are absent in hosts while being essential to the *Trypanosoma* and *Leishmania*’s survival. In contrast, the fact that mammalians own GR instead of TryR favours the specificity of a potential lead compound. Regarding the structural differences between TryR and GR, the substrate of TryR is bigger and is positively charged at physiological pH, whereas GSSG is negatively charged [116]. Considering all the advantages pointed out, these enzymes are validated as promising therapeutic targets for the development of more specific and safer inhibitors. Noteworthy, the available chemotherapy arsenal for treating these infections includes few drugs, which prove increasingly unsatisfactory mainly due to emerging resistance. Major drawbacks such as their weak efficacy, high toxicity, high cost, and inadequate mode of administration [117] boosted the need to find novel drugs that can effectively treat patients affected by these diseases [6].

## 5. Inhibitors of the TryR and TryS Enzymes

Efforts envisioning the development of novel anti-trypanosomatids agents have led to some advancement. Notably, the X-ray crystal structures of TryR from *T. brucei* and *T. cruzi* have been solved, showing an 84.0% amino acid homology and a 100.0% sequence similarity for the active site residues [118]. Moreover, amino acid sequence alignment showed that *Leishmania mexicana* TryR shares 45.0% and 91.0% identity with *T. cruzi* TryR and *Leishmania infantum* TryR, respectively [119]. Regarding TryS, studies revealed that *Leishmania major* TryS and *Leishmania donovani* TryS have an homology of 95.0%, whereas *T. brucei* TryS shares 72.0% of similarity with *T. cruzi* TryS [120]. All these structural findings denote a high amino acid homology among TryS and TryR from various species of *Leishmania* and *Trypanosoma*, suggesting the possibility of developing a standard inhibitor, effective against all trypanosomatids. Noteworthy, novel molecules should reduce the enzymatic activity by at least 90.0% to achieve the status of lead compound. This requirement assumes that impairment of the parasite’s redox viability only occurs when the IC_50_ values are within the low micromolar range [6,118].

### 5.1. Trypanothione Synthetase Inhibitors

Although TryS inhibitors have received less attention than TryR inhibitors, their discovery confirmed TryS as an excellent therapeutic target. In silico studies led to the identification of potential TryS inhibitors [112,121]. The compounds can be divided into substrate or transition stage analogues and other natural or synthetic compounds [122,123,124].

#### 5.1.1. Substrate or Transition State Analogues

Molecules that perform as substrate or transition state analogues are capable of binding to the active site of TryS, blocking its function and, consequently, reducing the rate of T(SH)_2_ biosynthesis from its polyamine precursors. Among several novel molecular entities investigated, 7,12-dihydrobenzo [2,3]azepino [4,5-b]indol-6(5*H*)-one and derivatives, known as paullones, showed promising inhibitory activities against *L. infantum* TryS. Paullones are a class of ATP analogues that have been identified as protein kinase inhibitors, interacting with the ATP binding pocket [112,125].

**Figure 4:**
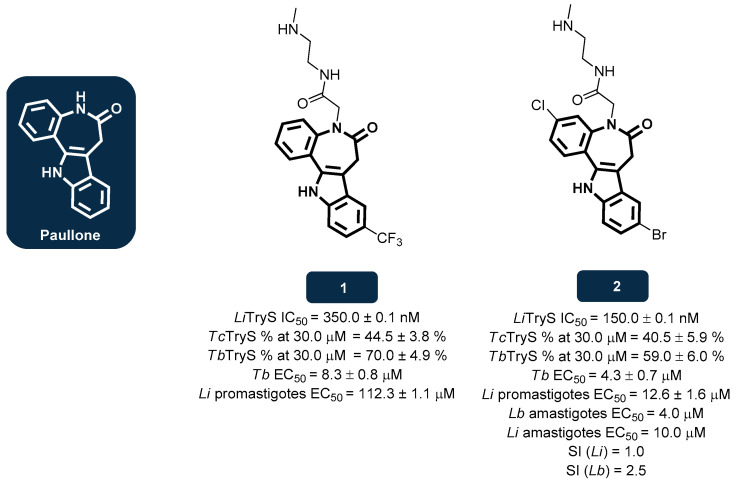
Structural representation of paullone derivatives **1** and **2**, with anti-TryS activity [128,129,130,131]. Legend: *Lb*, *L. braziliensis*; *Li*, *L. infantum*; *Li*TryS, *L. infantum* TryS; SI, Selectivity Index; *Tb*, *T. brucei*; *Tb*TryS, *T. brucei* TryS; *Tc*TryS, *T. cruzi* TryS.

Since paullones are known to disrupt the activity of several kinases, developing selective inhibitors against specific parasitic targets became a challenge. According to prior structural and mechanistic studies, the kinase inhibitory activity of paullones is related to the formation of a hydrogen bond involving the lactam nitrogen at position 5 [126], as shown in Figure 4. As such, it was assumed that N^5^-substituted paullones would become less active [127]. An investigation aiming to develop paullone analogues to specifically tackle parasitic diseases revealed that the addition of a *N*-[2-(methylamino)ethyl]acetamide side chain at the N^5^-position of 9-trifluoromethylpaullone [**1**] and 3-chlorokenpaullone [**2**] increased the inhibitory activity against *L. infantum* TryS (Figure 4). Both compounds exhibited high potency as inhibitors of *L. infantum* TryS, with **2** proving to be more potent than **1** (IC_50_ values of 150.0 nM and 350.0 nM, respectively) [128,129,130]. In further in vitro evaluations against *L. infantum* promastigotes, compounds **1** and **2** achieved EC_50_ values of 112.3 μM and 12.6 μM, respectively [129]. Compounds **2** and **1** were also assessed against *T. brucei*, achieving EC_50_ values of 4.3 μM and 8.3 μM, respectively [126,129]. Another set of paullones with different N^5^ substituents (acetic acid, its ester derivatives, or some acetamide derivatives) were synthesized and tested on *L. braziliensis* and *L. infantum*, unravelling novel derivatives with potent inhibitory properties that behave as non-competitive enzyme inhibitors. Compound **2** proved to be the best, achieving EC_50_ values of 4.0 μM and 10.0 μM towards *L. braziliensis* and *L. infantum* amastigotes, respectively [131]. However, selectivity studies revealed that **1** and **2** display high cytotoxicity towards mouse macrophages (cell line J774), with a selectivity index (SI) inferior to 10.0, clearly demanding selectivity improvements [129,131]. Assessments of **2** against the TryS enzymes of *T. cruzi* and *T. brucei* revealed moderate activity, with an inhibition of 40.5% and 59.0% at 30.0 μM, respectively. Compound **1** also exhibited a moderate activity, inhibiting TryS enzyme of *T. cruzi* and *T. brucei* by over 44.5% and 70.0% at 30.0 μM, respectively [128,129,130].

#### 5.1.2. Other Compounds

**Figure 5:**
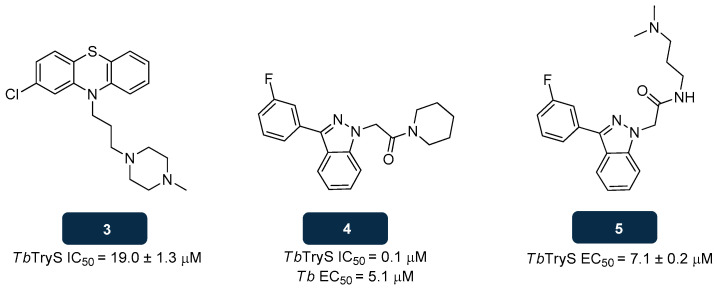
Structural representation of compounds **3**, **4**, and **5**, with anti-TryS activity [132,133]. Legend: *Tb*, *T. brucei*; *Tb*TryS, *T. brucei* TryS.

Recent efforts to develop novel anti-TryS candidates unravelled structures that proved to inhibit this enzyme through distinct mechanisms from those associated with the group of substrate or transition state analogues. Compound **3** (prochlorperazine) (Figure 5) is an antiemetic drug introduced to treat chemotherapy-induced dizziness, known to act by blocking D2 dopamine receptors. This compound was identified from in silico high-throughput screening (HTS) studies and posteriorly assessed against *T. brucei* TryS, evidencing an IC_50_ value of about 19.0 μM. The indazole derivative **4** (Figure 5) also exhibited excellent efficacy toward *T. brucei* (EC_50_ of 5.1 μM)*,* with studies showing its inhibitory activity against TryS (IC_50_ of 140.0 nM). Further research revealed a decrease in trypanothione’s intracellular levels upon incubation of *T. brucei* with **4**, at 40.0 μM for 72 h, together with glutathione increase, corroborating TryS as the target of **4** [112,129,132]. The indazole compound **5** (Figure 5), an analogue of **4**, was also reported as a potent inhibitor of the recombinant TryS of *T. brucei*, exhibiting an EC_50_ value of 7.1 μM [112,132,133].

**Figure 6:**
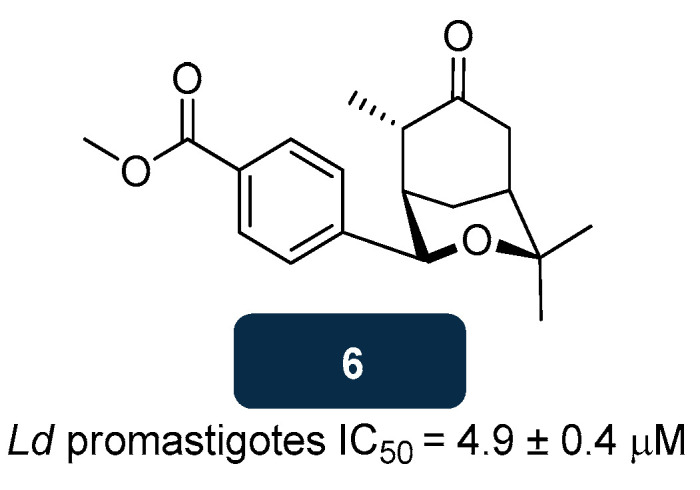
Structural representation of compound **6**, with anti-TryS and anti-TryR activity [134]. Legend: *Ld*, *L. donovani*.

Computational studies envisioning to identify *Leishmania* redox inhibitors unravelled oxabicyclonanone derivatives as potential competitive inhibitors of both TryR and TryS. From the library of compounds screened, compound **6** (Figure 6) exhibited the highest antileishmanial activity (IC_50_ of 4.9 μM) against *L. donovani* promastigotes. Mechanistic studies showed that **6** decreased threefold the T(SH)_2_ levels, while the levels of *Leishmania* TryS substrate increased threefold, leading to an increase in ROS levels within the parasite and, consequently, to apoptotic-like death. Similarly, when tested in downregulated *T. brucei* for TryS, **6** led to a fourfold increase of glutathione, together with a decrease of 86.0% in T(SH)_2_. Downregulation of TryR in *L. donovani* caused an 80.0% decrease in infection, compared to the untreated control, also consolidating TryR as a target. Cytotoxicity studies demonstrated the high selectivity of **6** for the parasite, without significant damage to the macrophage, even at concentrations above 100.0 μM [112,134].

**Figure 7:**
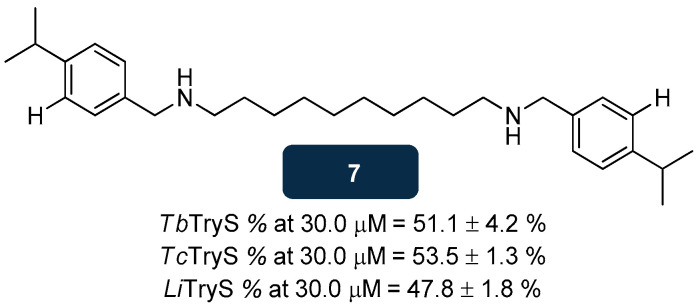
Structural representation of compound **7**, with anti-TryS activity [129]. Legend: *Li*TryS, *L. infantum* TryS; *Tb*TryS, *T. brucei* TryS; *Tc*TryS, *T. cruzi* TryS.

Diamine derivatives were evaluated as potential antiparasitic scaffolds, and the results highlighted compound **7** (Figure 7) as a TryS inhibitor transversal to several species. In vitro studies showed that **7** interferes with the T(SH)_2_ biosynthesis by inhibiting TryS of *L. infantum*, *T. cruzi* and T. *brucei* (inhibition at 30.0 μM of over 50.0%). Structure–activity relationship (SAR) data indicated that the incorporation of an isopropyl group in para position at two benzyl moieties of **7**, also including a 10-carbon linker, leads to improvement of up to 40.0% in inhibitory activity. Aiming to unravel the effect of **7** on the biological mechanisms of the target, *T. brucei* bloodstream forms were treated with 50.0 nM of **7,** and the results indicated a reduction of around 28% in T(SH)_2_, while the levels of GSH increased by 39.0% compared to the untreated cells. Moreover, through downregulation assessments, the toxicity of **7** was evaluated toward parasites lacking TryS. Theoretically, a compound targeting specifically TryS will exhibit an enhancement in cytotoxicity against parasites lacking TryS. Indeed, 98.0% of TryS-depleted parasites showed cytotoxic effects upon treatment with 100.0 nM of **7**, while the observed cytotoxicity towards wild type parasitic cells, i.e., with normal levels of TryS, was only about 76.0% [112,129].

**Figure 8:**
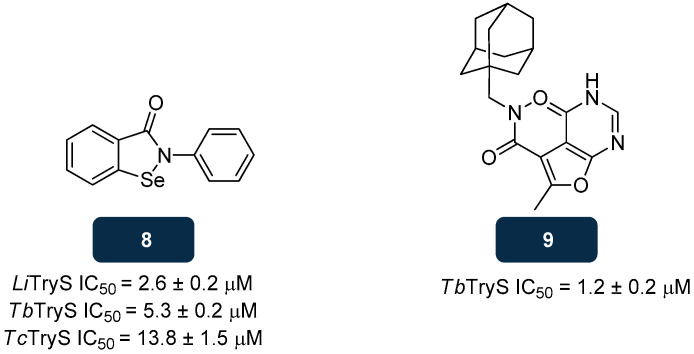
Structural representation of compounds **8** and **9**, with anti-TryS activity [135]. Legend: *Li*TryS, *L. infantum* TryS; *Tb*TryS, *T. brucei* TryS; *Tc*TryS, *T. cruzi* TryS.

Recent studies on a library of compounds screened against TryS led to the discovery of compound **8** (Figure 8), a selenorganic compound capable of inhibiting TryS of multiple species with IC_50_ values of 5.3 μM, 13.8 μM, and 2.6 μM, toward *T. brucei, T. cruzi,* and *L. infantum* TryS, respectively. Moreover, **8** displays anti-proliferative activity against parasites belonging to the *Trypanosoma* genus by inducing a rapid generation of intracellular oxidative stress. Its action on TryS causes an irreversible change in a highly conserved cysteine residue from the enzyme’s domain, acting as a slow-binding inhibitor. From the enamine-based compounds set under study, compound **9** (Figure 8) also emerged, a molecule comprising an adamantine moiety that evidenced efficacy against *T. brucei* TryS with an IC_50_ of 1.2 μM, as a non-covalent and non-competitive inhibitor [135].

**Figure 9:**
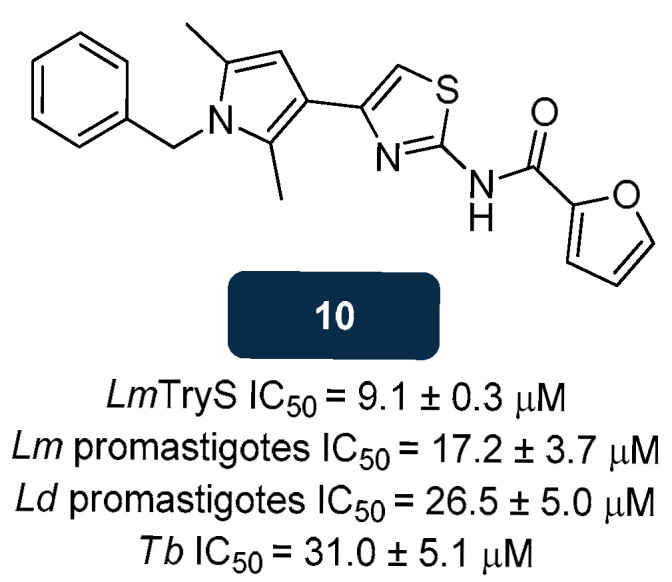
Structural representation of compound **10**, with anti-TryS activity [136]. Legend: *Ld*, *L. donovani*; *Lm*, *L. major*; *Lm*TryS, *L. major* TryS; *Tb*, *Trypanosoma brucei*.

Data from HTS assays revealed that compound **10** (Figure 9), containing a pyrrolothiazole-amide scaffold, could inhibit *L. major* TryS with an IC_50_ of about 9.1 μM. Regarding possible interactions of **10**, in silico studies revealed interactions of the thiazole’s nitrogen with the -OH functional group of Thr620, a residue that establishes a hydrogen bond with the ATP substrate. Arg328, involved in the GSH binding, establishes three hydrogen bonds with **10**, one with the amide moiety and the other two with the furan oxygen. However, **10** showed moderate activity against *L. major* and *L. donovani* promastigotes, as well as *T. brucei* (IC_50_ of about 17.2 μM, 26.5 μM, and 31.0 μM, respectively) [136].

**Figure 10:**
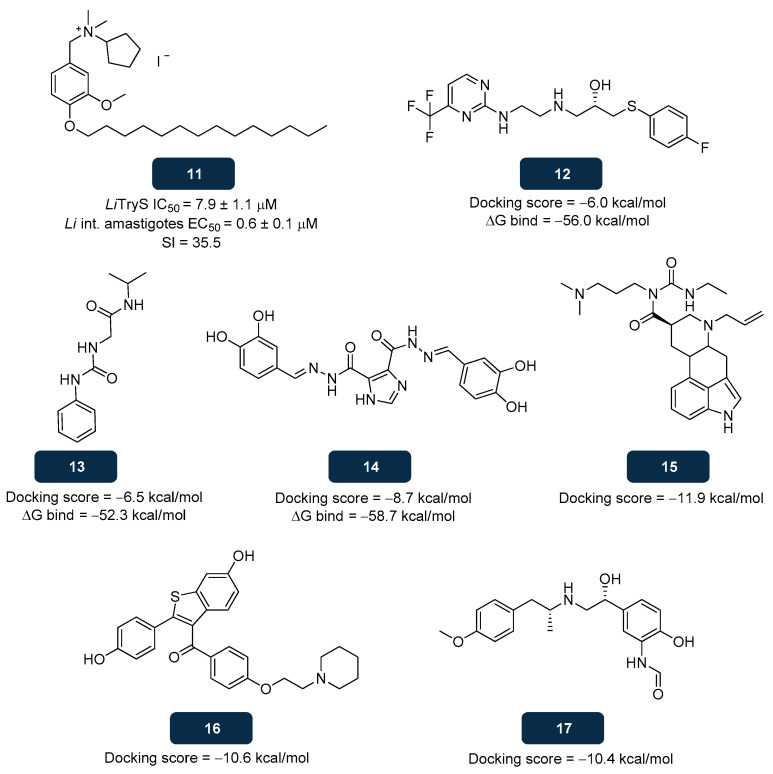
Structural representation of compounds **11**, **12**, **13**, **14**, **15**, **16**, and **17**, identified from in silico studies as potential TryS inhibitors [137,138,139]. Legend: *Li* int, Intracellular *L. infantum*; *Li*TryS, *L. infantum* TryS; SI, Selectivity Index; ΔG bind, Change in Gibbs free energy on binding to the target.

Overall, computational tools have emerged as predictive assays in the identification of leads, paving the way to further in silico and biological experiments, particularly in vitro and in vivo assays, to validate lead compounds’ pharmacokinetic and pharmacodynamic properties [121]. Recent computational studies led to the discovery of several novel TryS inhibitors. Alcón-Calderón et al. screened 144 molecules from a non-biased chemical library, identifying six potential anti-leishmanial structures. Subsequent in vitro studies established compound **11** (Figure 10) as the most potent anti-leishmanial candidate, with an EC_50_ value of 0.6 μM against *L. infantum* intracellular amastigotes, close to that obtained for miltefosine (EC_50_ of 0.7 μM), and a selectivity index of 35.5. The activity of **11** against *L. infantum* TryS was further analysed under saturating concentrations of all three substrates, ATP, glutathione, and spermidine, yielding an IC_50_ value of 7.9 μM. Additionally, it was demonstrated that the connection of **11** to the enzyme is affected by high concentrations of either glutathione or spermidine, indicating a competitive mode of inhibition, potentially occurring at the polyamine binding site in *L. infantum* TryS. Hence, the identical structural architecture of compound **11** and miltefosine may lead to comparable effects induced by both molecules. Impairments observed for both compounds include plasma membrane depolarisation, alterations in the mitochondrial inner membrane, and an increase in intracellular superoxide radical levels [137].

Another study focusing on structure-based virtual screening predicted that **12**, **13**, and **14** (Figure 10) were potential *L. donovani* TryS inhibitors. The binding strength of the protein-compound structure was evaluated by calculating the binding free energies. The results obtained for **12**, **13**, and **14** were −56.0 kcal/mol, −52.3 kcal/mol, and −58.7 kcal/mol, respectively, indicating a high binding affinity of the ligands toward TryS. Supporting the previous findings, docking studies demonstrated that both compounds interacted with residues of the TryS active site with docking scores of −6.0 kcal/mol, −6.5 kcal/mol, and −8.7 kcal/mol, respectively. Additionally, pharmacokinetic studies of the lead compounds confirmed their suitability as drug candidates [138].

Screening of a drug database containing 2000 compounds approved by the Food and Drug Administration, based on pharmacokinetic characteristics and Lipinski’s Rule of Five, led to repurposing existing drugs as novel TryS inhibitors. For molecular docking with TryS, only drugs that comply with the “Rule of Five” were selected. This effort led to the identification of **15**, **16**, and **17** (Figure 10) as promising TryS inhibitors, with docking scores of −11.9 kcal/mol, −10.6 kcal/mol, and −10.4 kcal/mol, respectively. Apart from filtering the number of molecules, docking studies revealed that hydrogen bonds with Ser230 and π–π interactions with Phe626 were common across the three compounds, highlighting their fundamental role in inhibiting *L. donovani* TryS function [139].

### 5.2. Trypanothione Reductase Inhibitors

In contrast to TryS, TryR has been extensively scrutinized, aiming to find better anti-trypanosomatids that can effectively inhibit this enzyme [140]. Efforts have led to the identification of several pharmacotherapeutic classes that block this enzyme in different ways, which may be classified into three categories: (1) trypanothione inhibitors, (2) NADPH inhibitors, and (3) catalytic inhibitors [6]. In parallel, compounds that act by mixed inhibition, by disassembly of the enzyme dimer, or that impair the enzyme from performing its normal function (Turncoat inhibitors) have also been described.

#### 5.2.1. Trypanothione Inhibitors

**Figure 11:**
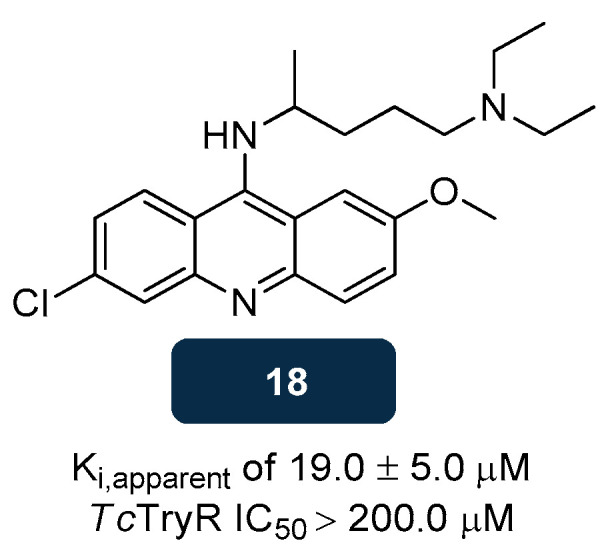
Structural representation of **mepacrine** (compound **18**), an antimalarial drug with anti-TryR activity [142,143]. Legend: K_i,apparent_, Apparent Inhibition Constant; *Tc*TryR, *T. cruzi* TryR.

Trypanothione inhibitors comprise a vast group of molecules that compete with TS_2_ for the TryR extensive active site. Mechanistically, the interaction of TS_2_ with TryR occurs through the spermidine scaffold, with the residues Tyr110, Glu18, Trp21, Ser109, and Met113 playing a major part in the mepacrine binding site (MBS). In GR, most residues are not conserved, including Glu18 (Ala34 in GR), Trp21 (Arg37), Ser109 (Ile113), and Met113 (Asn117), which underlies the selectivity of TS_2_ toward TryR [118,141]. Most molecules known as TryR inhibitors block the MBS, as in the case of the antimalarial drug **mepacrine** (compound **18**), a tricyclic compound (Figure 11) capable of inhibiting competitively *T. cruzi* TryR with an apparent inhibition constant (K_i,apparent_) of 19.0 μM [142]. Despite **18**′s weak TryR inhibitory capacity (IC_50_ > 200.0 μM) [143], X-ray studies revealed that it can occupy the hydrophobic cleft of TryR, the MBS, establishing three main interactions that involve the nitrogen of the acridine ring and Met113, the chlorine and Trp21, and the methoxy group and Ser109 [142]. Besides explaining the selectivity of mepacrine to TryR, this discovery showed that new effective and selective TryR inhibitors should have a hydrophobic core linked to an alkylamino chain as essential structural features [112,123].

**Figure 12:**
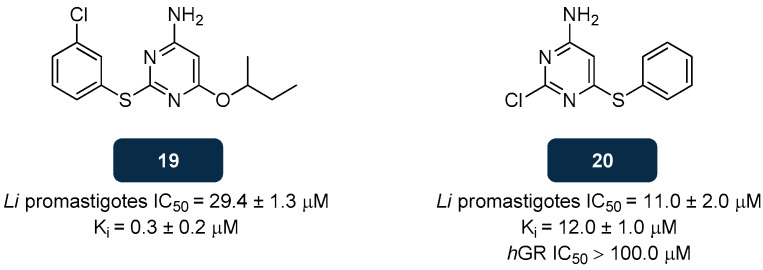
Structural representation of diarylsulphide derivatives **19** and **20**, with anti-TryR activity [144,145]. Legend: *h*GR, Human Glutathione Reductase; K_i_, Inhibition Constant; *Li*, *L. infantum*.

The activity of **18** raised the interest in tricyclic compounds as scaffolds for designing and developing new antiparasitic compounds through a rational target-based approach, yet their weak antiparasitic activities and neurological side effects hindered the identification of a lead capable of inhibiting TryR with good potency, low side effects, and high selectivity. Several researchers focused on addressing novel chemotypes as TryR inhibitors, and from these efforts diarylsulphides emerged, either by directly binding to the catalytic area of this enzyme or near it, and by binding to the NADPH-binding site, thus inhibiting the reduction of TS_2_ into T(SH)_2_. From this group compounds **19** and **20** (Figure 12) were selected, achieving moderate micromolar antileishmanial activity against *L. infantum* promastigotes, with IC_50_ values of 29.4 μM and 11.0 μM, respectively. Studies performed on these compounds revealed that **19** promotes the oxidation of NADPH by competitively inhibiting the binding of trypanothione to TryR. The inhibition constant (K_i_) for **19**, calculated from the Dixon plot analysis, was 0.3 μM, six times inferior to the K_i_ of the active form of antimonials Sb(III) (1.5 μM), the control drugs. However, the value of K_i_ for **20** was 12.0 μM, much higher than that for **19** and even higher than that of Sb(III). The intracellular trypanothione concentration was measured, showing a 33.2% decrease in T(SH)_2_ in parasites treated with **20** and 38.5% after treatment with **19** [144,145]. Compound **20** was also tested on human glutathione reductase (*h*GR), and its ineffectiveness (IC_50_ higher than 100.0 μM) indicates selectivity toward TryR [145].

**Figure 13:**
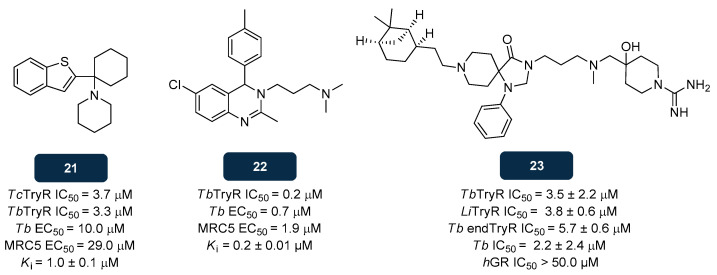
Structural representation of leads with anti-TryR activity; **21**, **22,** and **23** [146,149,150]. Legend: *h*GR, Human Glutathione Reductase; K_i_, Inhibition Constant; *Li*TryR, *L. infantum* TryR; *Tb*, *T. brucei*; *Tb* endTryR, Endogenous *T. brucei* TryR; *Tb*TryR, *T. brucei* TryR; *Tc*TryR, *T. cruzi* TryR.

HTS studies conducted by Patterson et al. [146] led to the identification of novel *T. cruzi* TryR inhibitors, from which the benzothiophene derivative **21** (Figure 13) was selected. Assessment of its in vitro inhibitory activity on *T. cruzi* and *T. brucei* TryR revealed a remarkable potency, with an IC_50_ of 3.7 μM and 3.3 μM, respectively [146]. Additionally, **21** was evaluated against the bloodstream form of *T. brucei*, exhibiting an EC_50_ value of 10.0 μM The safety of **21** was also scrutinized through evaluation of its activity against mammalian MRC5 cells, affording an EC_50_ of 29.0 μM, indicative of poor selectivity. Kinetic studies indicated that **21** acts as a competitive inhibitor of TryR, with a K_i_ value of 1.0 μM [146,147]. Gathered structure–activity relationship (SAR) data supported **21** optimisation. It was found that the binding affinity toward *T. brucei* TryR as well as the antitrypanosomatid activity could be optimised by adjusting the indole *N*-substituent and by introducing a propargylic substituent in position 4 of the thiazole moiety. These efforts yielded five inhibitors showing K_ic_ values in the sub-micromolar range [148].

In addition to **21** and analogues, Patterson’s team explored other chemical skeletons that could fulfil the criteria of a lead TryR inhibitor. Such efforts led to compounds bearing a 3,4-dihydroquinazoline core with inhibitory activity for both *T. cruzi* growth and TryR. SAR studies on this chemotype led to the selection of the C4-substituted compound **22** (Figure 13). This compound exhibited the best inhibitory activities for both *T. brucei* parasite (EC_50_ of about 0.7 μM) and TryR (IC_50_ of about 0.2 μM) [149]; however, it required further optimisation to improve selectivity, since the EC_50_ value against mammalian MRC5 cells was found to be of 1.9 μM. Studies of the TryR-**22** complex allowed gathering more detailed SAR information, revealing the insertion of a methyl group on the toluene substituent at C4 as the trigger for a fourfold improvement in potency. It was proposed that the methyl group is essential to position **22** into the active site, favouring hydrophobic interactions between the phenyl group of **22** and the Tyr110 residue. These results were consolidated by data from kinetic assessments, leading to the identification of compound **22** as the most potent analogue against TryR, with a K_i_ of about 0.2 μM [149].

Screening of a collection of 3097 antitrypanosomatid compounds led to the identification of the spiro derivative **23** (Figure 13) as a potential inhibitor of *T. brucei* and *L. infantum* TryR, with IC_50_ values of 3.5 μM and 3.8 μM, respectively. It was proposed that **23** competes with the enzyme’s substrate, TS_2_. Further studies assessed the potential of **23** as inhibitor of the endogenous *T. brucei* TryR activity and its anti-proliferative activity toward *T. brucei* parasites, showing that compound **23** effectively inhibits endogenous TryR and *T. brucei* parasites, with IC_50_ values of 5.7 μM and 2.2 μM, respectively. Selectivity evaluations confirmed the safety of **23**, as it exhibited an IC_50_ above 50.0 μM toward *h*GR [150]. The X-ray structure of the complex *T. brucei* TryR-**23** revealed interactions between compound **23** and the residues Trp21, Met113, and Tyr110, located in the MBS [150].

**Figure 14:**
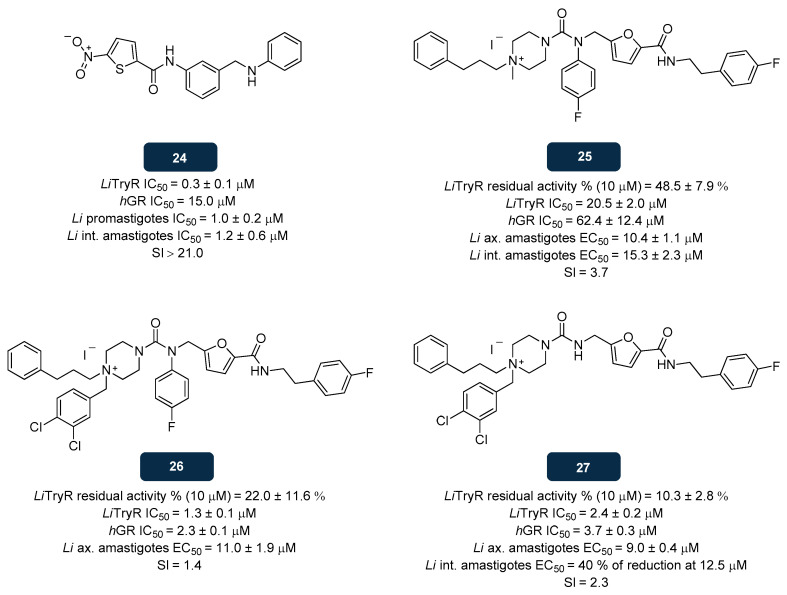
Structural representation of leads with anti-TryR activity; **24**, **25**, **26**, and **27** [141,151]. Legend: *h*GR, Human Glutathione Reductase; *Li*, *L. infantum*; *Li* ax amastigotes, *L. infantum* axenic amastigotes; *Li* int, Intracellular *L. infantum*; *Li*TryR, *L. infantum* TryR; SI, Selectivity Index.

HTS assays also unravelled nitro-substituted heterocycles as inhibitors of TryR by binding to the TS_2_ cavity, leading to the synthesis of several 5-nitrothiophene-2-carboxamides, screened as potential antileishmanial drugs. Among the molecules of this chemotype investigated, the amide-linked nitrothiophene-benzylamine conjugate **24** (Figure 14) emerged with sub-micromolar inhibitory activity against *L. infantum* TryR (IC_50_ of 0.3 μM), together with an IC_50_ of 15.0 μM on *h*GR, demonstrative of its low cytotoxicity. The same authors also selected compound **24** upon testing this compound against promastigotes and intracellular amastigote forms of *L. infantum,* obtaining IC_50_ values of 1.0 μM and 1.2 μM, respectively, associated with a selectivity index superior to 21.0 [151]. Docking studies revealed that compound **24** establishes H-bonds with Lys61′ and Glu467, also interacting with His461 and Pro462 by π−π stacking and hydrophobic interactions [151].

More recently, fragment-based drug discovery (FBDD) emerged as a promising approach for the discovery of novel TryR inhibitors. Succinctly, FBDD focuses on identifying low-molecular-weight structures (fragments) that target macromolecules. To develop hit compounds, these fragments are further enlarged or connected [152,153]. Following this approach, Exertier et al. [141] proposed compounds **25**, **26**, and **27** (Figure 14). The residual enzyme activity (%) of *L. infantum* TryR was assessed upon exposure to each of the three compounds at a concentration of 10.0 μM, and the results showed inhibitory activity of 48.5%, 22.0%, and 10.3% for compounds **25**, **26,** and **27**, respectively. The values of IC_50_ against *L. infantum* TryR were determined. Compounds **26** and **27** afforded IC_50_ of about 1.3 μM and 2.4 μM, respectively, whereas compound **25** exhibited lower *L. infantum* TryR activity, with IC_50_ of 20.5 μM. Despite their potency, the selectivity index (below 3) of these compounds revealed toxicity problems attributed to their non-selective action (*h*GR IC_50_ of 62.4 μM, 2.3 μM, and 3.7 μM for compounds **25**, **26**, and **27**, respectively). The in vitro antileishmanial activities of the three compounds against *L. infantum* axenic and intramacrophage amastigotes were evaluated. When tested against axenic amastigotes, compounds **27, 25,** and **26** exhibited EC_50_ values of 9.0 μM, 10.4 μM, and 11.0 μM, respectively. Compound **25** was also tested against intracellular amastigotes, achieving an EC_50_ of 15.3 μM. Unfortunately, compounds **25**, **26,** and **27** exhibited SI values in the 1.4–3.7 range, demonstrating low selectivity toward the parasite. The binding mode of compounds **25**, **26**, and **27** with TryR was elucidated through X-ray crystallography. It was observed that compounds **25**, **26**, and **27** establish interactions with three distinct areas of TryR, MBS, γ-Glu site (near to the catalytic site and composed of His461′, Glu466′, and Glu467′ residues), and the Z-site (an additional hydrophobic sub-pocket site near the MBS). Noteworthy, all these three areas are conserved between the *Leishmania* and *Trypanosoma* parasites. It could be postulated that the ethyl-*p*-fluorophenyl moiety docks all compounds to the Z-site, while the dichlorobenzyl and phenylpropyl scaffolds interact with Trp21 of the MBS site through π-bonding. In further SAR optimisations, the inhibitory activity could be enhanced by including extended groups that will target MBS, linkers with n superior to 3, and *N*-alkylation of the piperazine moiety. It was proposed that the incorporation of bulkier and charged moieties that target the Z-site may also be beneficial [141].

#### 5.2.2. Catalytic Inhibitors

Catalysis mediated by TryR implicates four specific residues on the inner of the active site: the redox cysteines Cys52 and Cys57, and the bases His461′ and Glu467′. The process of reducing TS_2_ to T(SH)_2_, mediated by TryR, is driven by electron transfer from NADPH through FAD, breaking the disulphide bridge, Cys52-Cys57, into its dithiol form, Cys52 and Cys57 [154]. It is known that pentavalent antimonials achieved the status of first-line treatment for leishmaniases by their efficacy in inhibiting several leishmanial targets, including TryR, through the coordination with Cys52, Cys57, and His461 residues [155]. The observed ability of organometallics to anchor Cys52 and Cys57 residues boosted recent studies directed to the development of complexes based on different metals [6].

Baiocco et al. [156] demonstrated the enhanced effectivity of silver nanoparticles (AgNPs) over antimonials through a similar mode of action. Their study shows that, like for antimonials, the TryR-Ag[0] complex establishes silver interactions with the catalytic trio of residues Cys52, Cys57, and His461. Evaluation of the in vitro antileishmanial efficacy of AgNPs towards *L. infantum* promastigotes and intracellular amastigotes afforded IC_50_ values of 2.2 μM and 1.8 μM, respectively [156].

**Figure 15:**
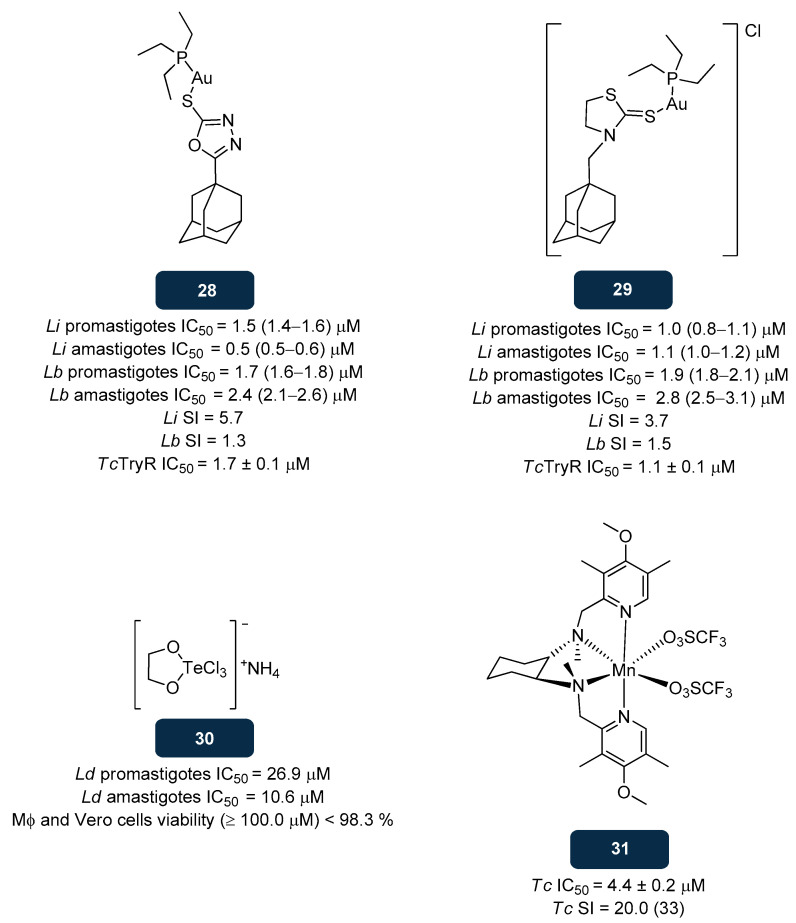
Structural representation of compounds **28**, **29**, **30**, and **31**, with proven anti-TryR activity [158,159,160]. Legend: *Lb*, *L. braziliensis*; *Ld*, *L. donovani*; *Li*, *L. infantum*; SI, Selectivity Index; *Tc*, *T. cruzi*; *Tc*TryR, *T. cruzi* TryR; Mϕ, macrophages.

Gold-based drugs have demonstrated a wide range of medicinal properties, including in the field parasitic infections [157]. In the search for novel TryR inhibitors, complexes **28** and **29** (Figure 15) stood out due to their potency against promastigotes and intracellular amastigotes of *L. braziliensis* and *L. infantum* [158]. In vitro studies of compounds **28** and **29** against promastigotes and intracellular amastigotes of *L. braziliensis* yielded IC_50_ values of 1.7 μM and 2.4 μM, respectively, for **28**, and 1.9 μM and 2.8 μM, respectively, for **29**. When tested against *L. infantum* promastigotes and amastigotes, **28** exhibited IC_50_ values of 1.5 μM and 0.5 μM, respectively, while **29** exhibited IC_50_ values of 1.0 μM and 1.1 μM, respectively. Overall, for both species and forms, IC_50_ values of **28** and **29** were within a low micromolar range, but the selectivity index gave poor results (below 10). Evaluations of the **28** and **29** inhibitory activity towards *T. cruzi* TryR were also conducted, revealing IC_50_ values in the low micromolar range (1.1–1.7 μM). Collected information from mechanistic studies indicated that TryR inhibition is mediated by Au(I) complexes, inducing ROS production and resulting in high levels of oxidative stress that will culminate in *Leishmania’s* apoptosis. A regimen comprising **29** and miltefosine was administered orally, over 13 days, to BALB/c mice infected with *L. amazonensis*. By the end of the treatment, the mean lesion size had decreased by 45% with miltefosine and by 65% with miltefosine and **29**, combined with a parasite burden reduction. From a pharmacokinetic point of view, gold complexes expressed high affinity to serum albumin, signalising the increased drug’s half-life. SAR insights suggested that a triethylphosphine-based scaffold merged with an adamantyl moiety boosts lipophilicity, supporting the passage of **28** and **29** across the cell membrane and privileging antileishmanial activity [158].

Some tellurium-based compounds with anti-tumoral potency have shown potential in the field of parasitic infections. The tellurium-based complex **30** (Figure 15), a non-toxic immunomodulator currently in phase II clinical trials for external genital warts and acute myeloid leukaemia, and in phase I clinical trial for patients with AIDS or AIDS-related complex, was also assessed against promastigotes and intracellular amastigotes of *L. donovani*, showing moderate potency against both forms (IC_50_ of 26.9 μM and 10.6 μM, respectively) [159]. Cytotoxicity experiments with **30** on macrophages demonstrated a viability of up to 98.3% at a drug concentration superior to 100.0 μM, revealing its safety. Studies on the mode of action of **30** ensued, firstly by detection of phosphatidylserine externalisation in the membrane of *Leishmania* promastigotes through Annexin-V/PI staining, followed by treatment with **30**. The outcomes indicated an increase in the number of cells that were both Annexin V- and PI-positive, of 32.8% (24 h), 66.6% (48 h), and 78.1% (72 h), compared to the positive control (62.6%) promastigotes treated with 4.0 mM H_2_O_2_. Such findings pointed to the ability of **30** to kill *Leishmania* through apoptosis induction, which prompted further studies aimed to gather deeper information on the mechanisms involved. Indeed, intracellular ROS production and impaired Ca^2+^ homeostasis signal the process of apoptosis in promastigotes. Applying the probe H_2_DCFDA, it was possible to identify 15.2% (3 h) and 61.4% (18 h) of ROS production after treatment with **30**. On the other hand, levels of Ca^2+^ in the *Leishmania* parasite increased from (48.0−54.0 nM) to 290.0 nM after 48 h of treatment with **30**. Furthermore, in vivo studies demonstrated the capacity of **30** (4.0 mg/kg) to eliminate totally the organ parasite load from *L. donovani*-infected Balb/c mice, and 93.0% from infected hamsters. Moreover, to confirm the mechanistic data gathered from in vitro studies, splenocytes were collected following treatment with **30**, showing 28.5% of ROS production after three weeks and 61.4% after five weeks. Aligned with these mechanistic findings, **30** interacts with Asp327, Met333, and Tyr198, highlighting both cysteines Cys52 and Cys57 of the catalytic site, as observed for other organometallic compounds already described [159].

Other organometallic compounds based on less scrutinised metals also contributed to the expansion of TryR inhibitors. The manganese-based complex **31** (Figure 15) inhibits trypomastigote forms of *T. cruzi* with an IC_50_ of 4.4 μM, exhibiting an SI of 20.0. It also exhibits a high TryR inhibitory activity, reaching 72.0% of TryR inhibition at 100.0 μM. In vivo studies conducted in female BALB/c mice showed a cure rate of 50.0% [160].

#### 5.2.3. NADPH Inhibitors

Apart from the crucial implication of cysteines 52 and 57 in reducing TS_2_, TryR is a NADPH-dependent flavoenzyme. As mentioned, the action of TryR is sparked via the action of Nicotinamide Adenine Dinucleotide Phosphate Hydrogen (NADPH) by providing two electrons to the cofactor FAD, followed by its transfer to TryR disulphide Cys57-Cys52. Then, the deprotonated Cys52, through the couple His461′-Glu466′, attacks the disulphide bridge of TS_2_, forming a mixed disulphide protein-substrate. Ultimately, the attack of Cys57 on Cys52 allows the break of the disulphide bridge, forming and liberating the reduced substrate, T(SH)_2_. NADPH anchors to TryR at a specific location, known as the NADPH-binding domain, this representing a potential therapeutic target for TryR inhibitors. In addition to the diarylsulphide derivatives **19** and **20**, mentioned in Subtopic 7.2.1, other antileishmanial compounds have been conceived as NADPH inhibitors [144,161].

**Figure 16:**
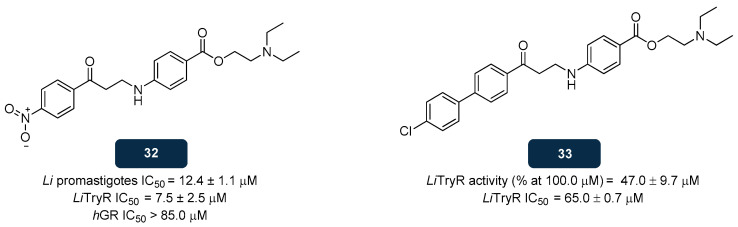
Structural representation of compounds **32** and **33**, with anti-TryR activity [161]. Legend: *h*GR, Human Glutathione Reductase; *Li*, *L. infantum*; *Li*TryR, *L. infantum* TryR.

As in previous works, the HTS assay screened over 100,000 small molecules to identify novel NADPH-competitive inhibitors. These efforts led to the identification of compound **32** (Figure 16), and subsequent in vitro evaluations against *L. infantum* promastigotes revealed its moderate activity (IC_50_ of 12.4 μM). Compound **32** was also tested against *L. infantum* TryR and *h*GR, exhibiting IC_50_ of 7.5 μM and 85.0 μM, respectively, indicating selectivity. Further studies revealed that compound **32** competes for NADPH but does not affect TS_2_ kinetics. The *L. infantum* TryR-**32** X-ray structure was solved, demonstrating that compound **32** binds to the NADPH cavity entrance, establishing several weak electrostatic interactions with Arg228, Gly197, Tyr221, Asn254, and Arg222. Undoubtedly, the binding site of NADPH comprises three essential residues (Arg222, Arg228, and Tyr221) ensuring the connection of the NADPH phosphate group to the enzyme, and it was uncovered that compound **32** displaces Arg 222 and Arg 228, thus preventing the connection of the cofactor to its binding site [162]. Given compound **32**′s promising results, new 3-amino-1-arylpropan-1-one derivatives were designed and synthesised, with a view to decrease the intrinsic toxicity caused by the nitro moiety. Among the small library of compounds disclosed, compound **33** (Figure 16) stood out, despite presenting only moderate activity (*L. infantum* TryR inhibitory activity of 47.0% at 100.0 μM, and an IC_50_ value of 65.0 μM). Docking studies indicate that compound **33** interacts with Arg228, Tyr221, and Gly195 residues. In contrast, compound **32** forms a hydrogen bond between its carbonyl group and Arg228, while compound **33** adopts a conformation that prevents it from forming. This difference may account for the lower *L. infantum* TryR-inhibiting potency of compound **33** compared to compound **32**. SAR hypotheses suggest that maintaining the ester linker and the 4-chlorophenyl substituent should be considered along with the preparation of further 3-amino-1-aryl propanone derivatives [161].

#### 5.2.4. TryR Dimer Disruptors

TryR is a homodimeric enzyme consisting of two identical subunits, each capable of bearing one molecule of TS_2_ and one molecule of NADPH. Given this attribute, researchers have scrutinised how TryR dimerization occurs to explore novel approaches for conceiving a new class of TryR inhibitors. In this respect, the dimerization analysis of *L. infantum* TryR interface underlined residue Glu436 as paramount for preserving the structural dimer integrity. Noteworthy, developing inhibitors for protein–protein interactions (PPIs) is considered quite challenging, since these interfaces generally miss well-defined binding pockets. In this sense, it was acknowledged that certain short linear and cyclic peptides, derived from an α-helix structure transiting from Pro435 to Met447 residues, significantly impair enzyme dimerization and TryR functionality [163].

**Figure 17:**
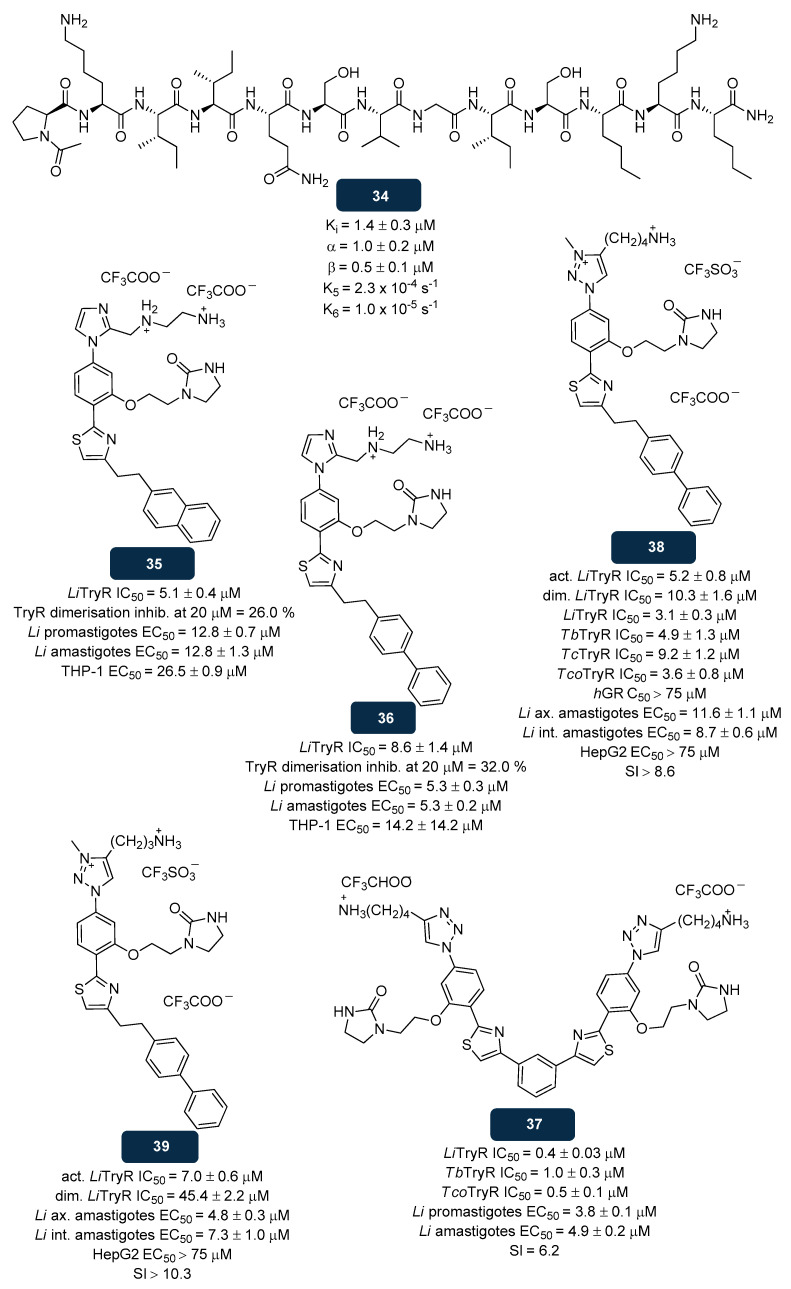
Structural representation of compounds **34**, **35**, **36**, **37**, **38,** and **39**, with proven anti-TryR activity [163,164,165,166]. Legend: α, Inhibitor’s binding affinity to the free enzyme compared to its binding affinity to the enzyme-substrate complex; act. *Li*TryR IC_50_, Inhibitory activity against *L. infantum* TryR oxidoreductase; β, Catalytic activity fraction between the enzyme–substrate–inhibitor complex to the enzyme–substrate complex; dim. *Li*TryR IC_50_, Inhibitory activity through monomer displacement of *L. infantum* TryR; *h*GR, Human Glutathione Reductase; K_i_, Inhibition Constant; k*_n_*, First-order constant number *n*; *Li*, *L. infantum*; *Li* ax. amastigotes, *L. infantum* axenic amastigotes; *Li* int. amastigotes, *L. infantum* intracellular amastigotes; *Li*TryR, *L. infantum* TryR; SI, Selectivity Index; *Tb*TryR, *T. brucei* TryR; *Tc*TryR, *T. cruzi* TryR; *Tco*TryR, *T. congolense*.

Highlighting that TS_2_ levels can exceed its Michaelis constant (K_m_) during oxidative stress events, TryR inhibitors with K_i_ values in the micromolar range are deemed unsuitable for combating trypanosomatids. Despite that, TryR is solely functional in its dimeric state. Bearing this in mind, the investigation for non-competitive TryR inhibitors is particularly attractive, since their activity remains unaffected by the variation of TS_2_ levels. Lucio et al. [164] undertook a deep kinetic study focused on non-competitive inhibitors of *L. infantum*-TryR that mimic the α2 helix of the *L. infantum* TryR dimerization interface. Compiled kinetic data point to a TryR inactivation over two consecutive steps, the binding occurrence followed by the TryR inactivation. Regarding the first step, compound **34** (Figure 17) [163] was identified as a non-competitive inhibitor binding both to the free enzyme and to the enzyme–substrate complex, with analogous affinities (α value of 1.0). Further insights predict the enzyme’s ability to catalyse the reaction when anchored to the substrate and the inhibitor [34], even though it operates at half the speed compared to the enzyme–substrate complex, as indicated by a β value of 0.5, along with a K_i_ value of 1.4 μM. Additionally, the curve of *L. infantum* TryR activity in the presence of **34** reveals a progressive drop of enzyme activity, characteristic of a slow-binding inhibitor. The second step of the process involves the slow isomerisation of the enzyme–inhibitor complex into an inactive enzyme. First-order constants k_5_ (2.3 × 10^−4^ s^−1^) and k_6_ (superior to the limit of 1.0 × 10^−5^ s^−1^) defined this step as a *pseudo-*irreversible inhibition mechanism [164].

Revuelto et al. [165] disclosed the synthesis of 15 compounds bearing pyrrolopyrimidines and 5-6-5 imidazole-phenyl-thiazole moieties. The advantage of these scaffolds lies in their ability to mimic the α-helix structure of TryR, with special orientation of the side chains for three crucial residues (Lys2, Gln5, and Ile9). From a chemical point of view, pyrrolopyrimidines and 5-6-5 imidazole-phenyl-thiazoles are favoured scaffolds due to their synthetic accessibility, conformational rigidity, and solubility in water, this facilitating oral availability. On the other hand, water is a polar solvent (dielectric constant at 25 °C of 78.4) that can interact with -N and -S heteroatoms incorporated in 5-6-5 imidazole-phenyl-thiazoles and pyrrolopyrimidine scaffolds through solvation, also stabilizing ionic groups. These phenomena could lead to desolvation penalties, impacting the energy balance. Following in vitro studies, the imidazole-phenyl-thiazole-based compounds **35** and **36** (Figure 17) emerged as the first nonpeptidic dissociative inhibitors of *L. infantum* TryR, affording IC_50_ values of 5.1 μM [35] and 8.6 μM [36] against *L. infantum* TryR oxidoreductase. Additionally, at a concentration of 20.0 μM, **35** and **36** exhibited TryR dimerization inhibition of 26.0% and 32.0%, respectively. When tested against promastigotes and amastigotes of *L. infantum*, **35** and **36** afforded EC_50_ values of 12.8 μM and 5.3 μM, respectively, for both forms. The compounds’ cytotoxicity was evaluated against the THP-1 cell line, revealing EC_50_ values of 26.5 μM [35] and 14.2 μM [36], indicative of a toxic profile. Overall, in vitro studies and molecular dynamics results revealed multiple binding sites for **35** and **36**. As shown by the solved crystal structure, their first binding site is the TryR catalytic site, but along with inhibiting the catalytic domain, their second binding site fits into the TryR dimerization interface. This interaction is facilitated by a bulky aromatic group, allowing compounds **35** and **36** to operate as dimer disruptors [165].

Besides the promising results gathered for the imidazole-phenyl-thiazole conjugates described above, Revuelto et al. [166] conducted studies to explore novel candidates as dimer inhibitors, based on imidazole replacement by a 1,2,3-triazole moiety. From the whole library of structures, the symmetrical triazole-based compound **37** (Figure 17) was the most potent toward TryR oxidoreductase of *L. infantum*, *T. brucei*, and *Trypanosoma congolense*, displaying IC_50_ values of 0.4 μM, 1.0 μM, and 0.5 μM, respectively. Compound **37** also inhibited *L. infantum* promastigotes and amastigotes with EC_50_ values of 3.8 μM and 4.9 μM, respectively, but exhibited a poor selectivity index of 6.2. Docking studies revealed hydrophobic interactions between the aromatic rings of compound **37** and TryR. In addition, the two terminal butylammonium groups establish coexisting salt bridges with Glu466/Glu467 and Glu466′/Glu467′, while the carbonyl groups of the two imidazolidinone moieties form hydrogen bonds with the protonated nitrogens of Lys61/Lys61′, the side-chain carboxamide of Gln68, and the NH_2_ of Ser433/Ser433′. Notably, **37** is considerably more potent than its monomeric analogue against both forms of *L. infantum*, indicating that the architecture of symmetric triazole-based compounds is effective in promoting more effective dimeric disruptors [166].

In 2022, Revuelvo et al. published a study detailing a series of novel triazole-phenylthiazole compounds identified as disruptors of TryR dimerization. Compounds **38** and **39** (Figure 17) stood out for further drug development, given their notable inhibitory activity against *L. infantum* TryR oxidoreductase, with IC_50_ values of 5.2 μM and 7.0 μM, respectively. Assessments conducted through *L. infantum* TryR monomer displacement revealed IC_50_ values of 10.3 μM for **38** and 45.4 μM for **39**. Furthermore, **38** exhibited potent inhibitory activity against TryR enzymes from *L. infantum*, *T. brucei*, *T. cruzi*, and *T. congolense*, achieving IC_50_ values lower than 10.0 μM. In contrast, assessment of **38** against *h*GR revealed an IC_50_ exceeding 75.0 μM, proving its selectivity and safety. Leishmanicidal activity tests on *L. infantum* axenic amastigotes revealed EC_50_ values of 11.6 μM and 4.8 μM for **38** and **39**, respectively. Further assessments against *L. infantum* intracellular amastigotes showed the effectivity of **38** and **39**, with EC_50_ values of 8.7 μM and 7.3 μM, respectively. It was also shown that the 1,2,3-triazolium salt **38** can reduce by 50.0% the non-protein thiol content of *L. infantum* promastigotes grown under mildly oxidative stress conditions in the presence of D,L-buthionine-(S,R)-sulfoximine (BSO), an inhibitor of γ-glutamylcysteine synthetase, a key enzyme that defines the rate of glutathione production [167,168].

#### 5.2.5. Mixed Inhibitors

Several chemical structures have been pinpointed as mixed inhibitors, due to their ability to bind to the enzyme regardless of whether it has already been affixed to the substrate [112,169,170,171]. Nevertheless, the affinity of these inhibitors may vary between the enzyme and the enzyme–substrate complex. As mentioned in the previous Subtopic 7.2.4., once an inhibitor exhibits the same affinity for the enzyme and the enzyme–substrate complex, it is categorised as a noncompetitive inhibitor [172,173].

**Figure 18:**
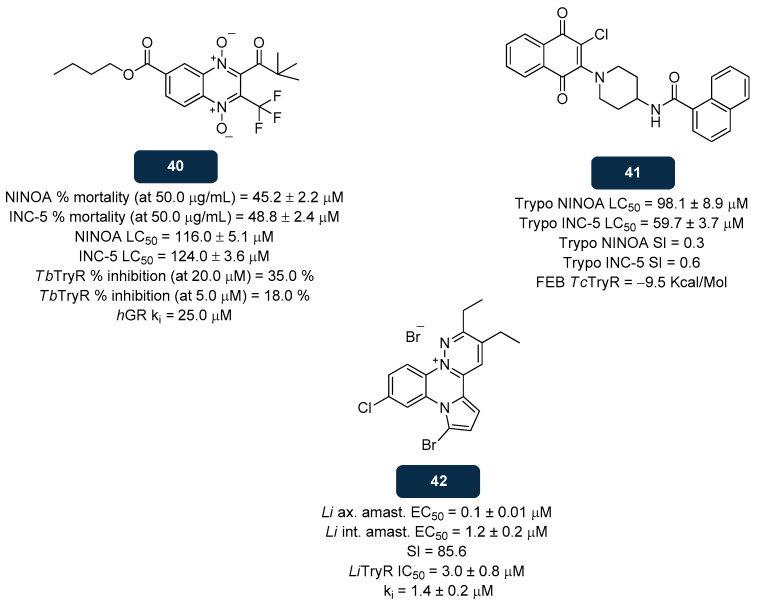
Structural representation of compounds **40**, **41**, and **42**, with anti-TryR activity [169,170,171]. Legend: FEB, Free Energy of Binding; *h*GR, Human Glutathione Reductase; K_i_, Inhibition Constant; *Li* ax. amast, *L. infantum* axenic amastigotes; *Li* int. amast, *L. infantum* intracellular amastigotes; *Li*TryR, *L. infantum* TryR; SI, Selectivity Index; *Tb*TryR, *T. brucei* TryR; Trypo, Trypomastigotes.

Prior research by González-González et al. [169] revealed the activity of novel n-butyl and isobutyl quinoxaline-7-carboxylate 1,4-di-*N*-oxide derivatives as TryR inhibitors against NINOA and INC-5 *T. cruzi* strains. Compound **40** (Figure 18), an n-butyl derivative with a trifluoromethyl group, achieved a mortality toward NINOA and INC-5 trypomastigotes strains of 45.2% and 48.8%, respectively, at a concentration of 50.0 μg/mL. Moreover, evaluations of the half-maximal lytic concentration (LC_50_) of **40** against NINOA and INC-5 trypomastigotes yielded LC_50_ values of 116.0 μM and 124.0 μM, respectively. Notably, the LC_50_ values obtained for **40** were lower than those achieved when using the reference drugs benznidazole (191.3 μM) and nifurtimox (139.4 μM). The inhibitory capacity of **40** against *T. cruzi* TryR was evaluated at varying concentrations and maintained the TS_2_ concentration of 44.0 μM. At a **40** concentration of 20.0 μM, the inhibition reached 35.0%, while at 5.0 μM, it was of 18.0%. Kinetically, the Lineweaver–Burk plot revealed a mixed-type inhibition of *T. brucei* TryR by **40**, with inhibitor constants K_i_ and K_i’_ of 11.4 μM and 60.8 μM, respectively. Furthermore, a Lineweaver–Burk plot was evaluated for *h*GR, describing **40** non-competitive inhibition. Unfortunately both enzymes showed similar K_i_ values for **40** (*h*GR of 25.0 μM), demonstrating its lack of selectivity as a TryR inhibitor. Docking studies indicated that large aromatic groups and protonable amines enhance the affinity of *T. cruzi* TryR inhibitors for the catalytic site. In line with docking findings, SAR analysis specified that aromatic substituents ameliorate the anti-trypanosomatid activity, particularly when functionalised at the 2-position [169].

Another study [170] unveiled novel naphthoquinone derivatives bearing nitrogen-based scaffolds merged with specific blocks, known for their effectivity against *T. cruzi*. Compound **41** (Figure 18) emerged as the most potent, with LC_50_ values of about 98.1 μM and 59.7 μM against trypomastigotes of *T. cruzi* NINOA and INC-5 strains, respectively. Notably, the LC_50_ values obtained for **41** were inferior to those obtained for benznidazole, the drug reference, in both strains. On the other hand, **41** did not achieve good selectivity index values for the trypomastigote forms (below 1.0 for both strains), revealing its toxicity. The free energy of binding (FEB) values for this series of TryR inhibitors were determined. Compound **41** exhibited one of the lowest FEB values, −9.5 Kcal/Mol, ascribed to substitution of the phenyl group by a naphthyl group. In line with the previous study, it was postulated that these findings emphasize the importance of large aromatic groups in forming interactions with the binding site. Compiled results from docking studies and kinetics experiments indicated that **41** is a mixed-type inhibitor of *T. cruzi* TryR, interacting at a domain distinct from the substrate-binding site. Docking studies with **41** were conducted on a hydrophobic region of *T. cruzi* TryR, the Z-site, revealing hydrophobic interactions of **41** with the residues Phe367, Pro371, Pro435, and Glu436, as well as hydrogen bonds with residues Gln69, His73, and Asn433 [170].

From a set of fifteen pyridazino-pyrrolo-quinoxalinium salts, compound **42** (Figure 18) was the most potent, with EC_50_ values of 0.1 μM and 1.2 μM, against *L. infantum* axenic and intracellular amastigotes, respectively, and an IC_50_ value of 3.0 μM against *L. infantum* TryR. Notably, **42** showed high selectivity, presenting an SI of 85.6. Given the promising results of **42** as inhibitor of both forms of *L. infantum* and TryR, further studies were conducted to unravel its mode of inhibition. In the presence of saturating concentrations of NADPH and variations of TS_2_, **42** acted as a noncompetitive inhibitor of TryR. However, in the inverse situation, when TS_2_ was saturated and NADPH varied, **42** behaved as an uncompetitive inhibitor. Overall, **42** acts as a subversive substrate for TryR, reaching a K_i_ for binding to the enzyme/substrate complex of about 1.4 μM [171].

#### 5.2.6. Turncoat Inhibitors (“Subversive” Substrates)

Turncoat inhibitors, also named “subversive” substrates, behave as uncompetitive inhibitors that bind to the enzyme–substrate complex instead of the free enzyme. These compounds are reduced by the enzyme in a single-electron step, forming a radical anion that reacts with oxygen to form highly reactive superoxide anion radicals (subversive substrates). These structures lead to oxidative stress, causing various types of damage to the parasite. During this process NADPH and O_2_ are consumed, which impairs the reduction of trypanothione disulphide and disrupts the parasite’s thiol–disulphide balance [112,174].

**Figure 19:**
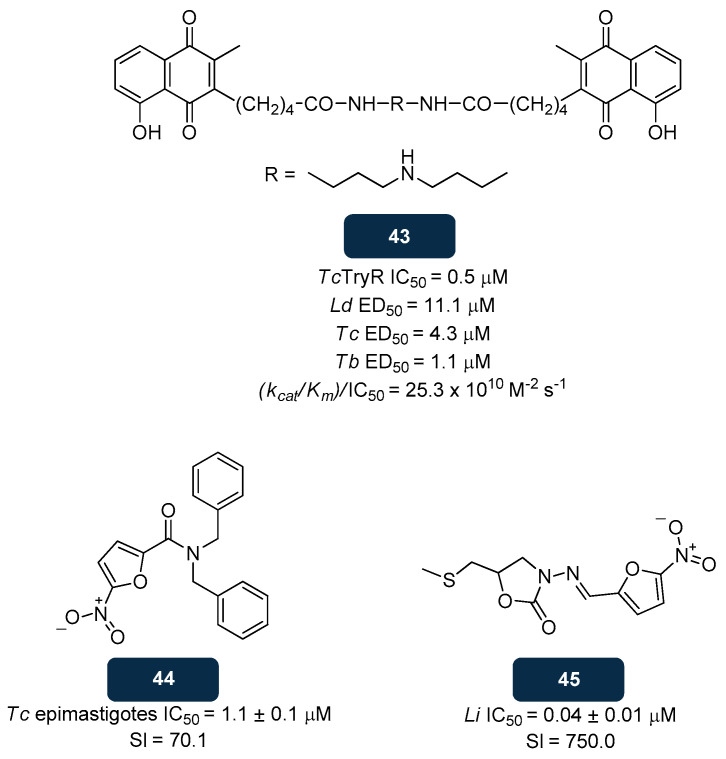
Structural representation of compounds **43**, **44**, and **45**, with anti-TryR activity [176,178,179]. Legend: k_cat_, Turnover number; K_m_, Michaelis-Menten Constant; *Ld*, *L. donovani*; *Li*, *L. infantum*; SI, Selectivity Index; *Tb*, *T. brucei*; *Tc*, *T. cruzi*; *Tc*TryR, *T. cruzi* TryR.

Naphthoquinone-based compounds emerged as turncoat inhibitors in the parasitic field. Indeed, reports exposed the activity of menadione (vitamin K3) and other related 1,4-napththoquinone derivatives against plasmodial thioredoxin and glutathione reductases [175]. Regarding trypanosomatid parasites, the synthesis and biological evaluations of 2- and 3-substituted 1,4-naphthoquinone-based compounds were uncovered. Compound **43** (Figure 19) arose as the most effective inhibitor of TryR in *T. cruzi*, displaying an IC_50_ value of about 0.5 μM when tested alongside 57.0 μM TS_2_. Notably, the **43′** IC_50_ value is 62 times lower than that of its parent compound, plumbagin, which exhibited an IC_50_ of 28.0 μM, also presenting a redox cycling of 201-fold at a concentration of 25.0 μM. Assessments of the median effective dose (ED_50_) against *L. donovani*, *T. cruzi*, and *T. brucei* achieved results in the low micromolar range (1.1–11.1 μM). Noteworthy, **43** displays effective trypanocidal and redox cycling activity, combined with good inhibitory potency of TS_2_ reduction. Further evaluation of the **43′** mode of inhibition shows its effectiveness as a turncoat inhibitor, with the highest (*k_cat_*/*K_m_*)/IC_50_ value of 25.3 × 10^10^ M^−2^ s^−1^, along with a favourable rate of redox cycling. To collect information about the mode of action of **43**, superoxide dismutase (SOD) was introduced in the presence of 12.5 μM of **43**. The results indicated a decrease of over 88.0% in the reduction rate of cytochrome c, proving the production of superoxide anion radicals through the reduction of **43** by TryR. Conversely, the rate of cytochrome c reduction in *h*GR was not significantly increased, showing only a 1.1-fold change, which confirms that *h*GR does not reduce **43** [176].

Nitrofuran derivatives have been shown to irreversibly inhibit TryR activity under anaerobic conditions through noncompetitive inhibition. In contrast, when oxygen (O_2_) is present, these derivatives act as turncoat inhibitors (uncompetitive inhibition), leading to several harmful effects on *T. cruzi* parasites, including inhibition of TS_2_ reduction, increased production of free radicals, and reduction in NADPH levels due to its frequent consumption during the conversion of O_2_ into superoxide (O_2_^−^) [112,177].

Novel 5-nitro-2-furoic acid analogues have emerged among uncompetitive inhibitors that act as turncoat inhibitors. The trypanocidal activity of these compounds was tested against *T. cruzi* epimastigotes. Compound **44** (Figure 19) stood out, with an IC_50_ of 1.0 μM and a selective index of 70.1, thus demonstrating selectivity toward the parasite. Kinetic studies on the inhibition of *T. cruzi* TryR by **44** demonstrated reversible uncompetitive inhibition concerning TS_2_. Previous findings were further supported by molecular docking studies, which indicated that **44** can bind directly to the *T. cruzi* TryR–TS_2_ complex. These studies also revealed that the rotational freedom of **44**, due to the methylene group of the benzyl moiety, confers flexibility, allowing the phenyl groups to align in parallel, establishing potential π interactions. Conversely, SAR’s evaluations indicated that the incorporation of a tertiary amine not belonging to a ring provides structural flexibility, which may contribute to the enhanced bioactivity of **44** [178].

A screen of 1769 compounds in ex vivo mouse splenocytes infected with an infrared-emitting *L. infantum* strain identified the nitrofuran derivative **45** (Figure 19), with the lowest IC_50_ of 0.04 μM, associated with an excellent SI value of 750.0. The in vivo efficacy of **45** was assessed in Balb/c mice infected with *L. donovani*. The administration of a daily dosage of 100.0 mg/kg body weight, over 10 days, resulted in a 78.2% reduction in in vivo bioluminescence. Conversely, **45** was highly effective against Balb/c mice infected with *L. major*, reducing the parasitic load below the detection limit and even surpassing Glucantime, the control drug [179]. Moreover, the combination of **45** with Miltefosine showed synergic antileishmanial effects for both in vitro and in vivo models [180].

## 6. Critical Analysis and Conclusions

As the private sector has increasingly deprioritised NTDs, initiatives that join academia, health organisations, the social sector, and industry have emerged as strategic approaches to advance NTD research. However, most academic research still relies on preliminary studies, as evidenced in this manuscript. Indeed, conducted research focused on discovering new hit compounds to target TryS or TryR mainly involved in vitro screening, with a very limited number of compounds progressing to more advanced studies. Budget constraints in academia are the primary reason to confine investigations to molecular in silico and in vitro screenings towards TryS and TryR, thus providing mostly proof of concept. Consequently, there is a critical need for further studies, to confirm in vivo efficacy, evaluate selectivity and toxicological parameters, and gather counter screening data, among other studies required to advance leads.

Major key factors highlight the potential of TryR and TryS as promising targets for development of inhibitors that can be developed into new drugs. TryS and TryR are unique to *Leishmania* and *Trypanosoma* parasites and play an instrumental role in the parasites’ ability to survive against oxidative stress, exhibiting traits that are comparable across species and genera. In fact, TryR and TryS exhibit a high degree of homology across trypanosomatid species, are instrumental for the survival of trypanosomatids, and feature specific druggable pockets (e.g., the NADPH binding site) that can be exploited in the design of selective inhibitors and further tuning of their pharmacological properties. Furthermore, these enzymes are absent in human hosts, minimising the risk of off-target effects and increasing the chances of selecting safer candidates. However, targeting the redox machinery enzymes, specifically TryS and TryR, poses significant challenges due to their remarkable efficiency and high turnover speeds. In fact, to impact the parasite’s survival, new therapeutic drugs must be able to reduce the enzyme’s activity by at least 90%. Ideally, a robust inhibitor should exhibit an IC_50_ value lower than 1 μM to be considered a promising lead compound, along with the search for effective treatments. Most of the structures presented in this review were subjected to screening studies; however, only some underwent further analyses. From a pharmacokinetic standpoint, lead compounds must meet ADME properties to be considered suitable hit candidates. For instance, compounds 12–17 were validated according to the Lipinski rule as anti-TryS, confirming their drug-like characteristics. In the case of TryR, gold complexes incorporating an adamantanone moiety and a triethylphosphine-based scaffold exhibit a high affinity for serum albumin, suggesting an increased drug half-life and enhanced antiparasitic effectiveness. Nevertheless, due to the limited data on this topic, further studies are still required to fully understand inhibitor–target interactions/modes of action, as well as to obtain further insight on the pharmacokinetic properties. The need of further studies is especially prominent regarding the TryS inhibitors.

The development of suitable molecular probes is also very relevant for obtaining deeper mechanistic insights regarding efficacy and selectivity of the molecules under study, since they offer the possibility of assessing if additional biological targets are affected. This strategy can be applied to the novel molecules under study to fight African Trypanosomiasis, leishmaniases, and Chagas Disease, and also the drugs in use to fight these NTDs, for which the mechanisms of action remain mostly elusive. In fact, a deeper knowledge of the mechanisms of action of the drugs in use could help in circumventing their limitations, for instance through drug optimisation strategies or by adjusting pharmacotherapeutics.

From a pharmacotherapeutic point of view, the combination of a TryS inhibitor with a TryR inhibitor could represent an effective way of controlling those parasitic diseases, by targeting different therapeutic targets that are essential to maintain parasites’ viability, thereby increasing efficacy. Also, combinations can effectively delay the loss of efficacy through selection for drug resistance. Approved drug combinations are known that take advantage of this type of synergism, where the compounds inhibit different enzymes involved in the same metabolic pathway. A well-known example is the antifolate combination of sulfamethoxazole with trimethoprim (known as co-trimoxazole), where sulfamethoxazole inhibits dihydropteroate synthase, and trimethoprim inhibits dihydrofolate reductase. By analogy, it is possible that the combination of a TryS inhibitor with a TryR inhibitor could be highly beneficial in the fight against infections caused by *Trypanosoma* and *Leishmania* species. This strategic approach requires further investigations.

## Data Availability

No new data were created or analysed in this study. Data sharing is not applicable to this article.
